# Retrograde movements determine effective stem cell numbers in the intestine

**DOI:** 10.1038/s41586-022-04962-0

**Published:** 2022-07-13

**Authors:** Maria Azkanaz, Bernat Corominas-Murtra, Saskia I.J. Ellenbroek, Lotte Bruens, Anna T. Webb, Dimitrios Laskaris, Koen C. Oost, Simona J.A. Lafirenze, Karl Annusver, Hendrik A. Messal, Sharif Iqbal, Dustin J. Flanagan, David J. Huels, Felipe Rojas-Rodríguez, Miguel Vizoso, Maria Kasper, Owen J. Sansom, Hugo J. Snippert, Prisca Liberali, Benjamin D. Simons, Pekka Katajisto, Edouard Hannezo, Jacco van Rheenen

**Affiliations:** 1 Department of Molecular Pathology, The Netherlands Cancer Institute, Amsterdam, The Netherlands; 2 Oncode Institute, the Netherlands; 3 Institute of Biology, University of Graz, Graz, Austria; 4 Institute for Science and Technology Austria, Klosterneuburg, Austria; 5 Department of Cell and Molecular Biology (CMB), Karolinska Institutet, Stockholm, Sweden; 6 Friedrich Miescher Institute for Biomedical Research (FMI), Basel, Switzerland; 7 Institute of Biotechnology, HiLIFE, University of Helsinki, Helsinki, Finland; 8 Molecular and Integrative Bioscience Research Programme, Faculty of Biological and Environmental Sciences, University of Helsinki, Helsinki, Finland; 9 CRUK Beatson Institute, Glasgow, UK; 10 Laboratory for Experimental Oncology and Radiobiology, Center for Experimental and Molecular Medicine, Cancer Center Amsterdam, Amsterdam Gastroenterology Endocrinology and Metabolism, Amsterdam University Medical Centers, Amsterdam, the Netherlands; 11 Institute of Cancer Sciences, University of Glasgow, Glasgow, UK; 12 Molecular Cancer Research, Centre for Molecular Medicine, University Medical Centre Utrecht, Utrecht, The Netherlands; 13 University of Basel, Basel, Switzerland; 14 Wellcome Trust-Cancer Research UK Gurdon Institute, University of Cambridge, Cambridge, UK; 15 Department of Applied Mathematics and Theoretical Physics, Centre for Mathematical Sciences, University of Cambridge, Cambridge, UK; 16 Wellcome Trust-Medical Research Council Cambridge Stem Cell Institute, Jeffrey Cheah Biomedical Centre, University of Cambridge, Cambridge, UK

## Abstract

Morphology and functionality of the epithelial lining differ along the intestinal tract, yet tissue renewal at all sites is driven by stem cells at the base of crypts^1–3^. Whether stem cell numbers and behaviour vary at different sites is unknown. Here, we show by intravital microscopy that despite similarities in the number and distribution of proliferative cells with an *Lgr5* signature, small intestinal (SI) crypts contain twice as many effective stem cells as large intestinal (LI) crypts. We find that, although passively displaced by a conveyor belt-like upward movement, SI cells positioned away from the crypt base can function as long-term effective stem cells due to Wnt-dependent retrograde cellular movement. By contrast, the near absence of retrograde movement in the LI restricts cell repositioning, leading to a reduced effective stem cell number. Moreover, upon suppression of the retrograde movement in the SI, the number of effective stem cells is reduced, and the rate of monoclonal conversion of crypts is accelerated. Together, these results show that effective stem cell number is determined by active retrograde movement, revealing a new channel of stem cell regulation that can be experimentally and pharmacologically manipulated.

## Introduction

The intestinal tract consists of multiple compartments with different functions, together ensuring digestion and uptake of nutrients, absorption of water and expelling remainders of food intake. The **small intestine** (**SI**) comprises villi that project into the gut lumen and crypts that invaginate into the mucosa. The **large intestine** (**LI**), comprising the caecum and colon, has a similar crypt architecture to the SI, but lack luminal-projecting villi. In both regions, mitotic stem cell zones form at the bottom of crypts with more differentiated epithelial cells positioned towards the gut lumen. The entire intestinal tube is lined with a single layer of epithelial cells that gets renewed every few days. This high turnover has been shown to be the consequence of dividing stem cells that compete neutrally for niche space at the base of each crypt^1,2^. Following each stem cell division, one cell becomes displaced upwards along the crypt-lumen axis to differentiate into specialized cells such as goblet cells and enterocytes. Markers, including **Leucine-rich repeat-containing G-protein coupled receptor 5** (**LGR5**), have been associated with stem cell activity^3^. However, such markers do not necessarily label all or exclusively cells that have the potential to form clones that persist over the long-term^4^–^8^, considered here as the defining property of **effective stem cells**. Using short-term intravital microscopy, we have previously shown that not every Lgr5^+^ cell has the same survival potential and therefore to act as a long-term effective stem cell^9^. Compared to Lgr5^+^ cells positioned at the base of crypts, Lgr5^+^ cells further away are more susceptible to passive displacement and subsequent loss, and therefore have a lower chance to form clones over the long-term^9^. However, the distance from the crypt base at which cells have a realistic chance to function as long-term stem cells, i.e. the number of effective stem cells, is currently unknown due to technical constraints in intravital microscopy that prohibit the same clone to be followed over several weeks. In addition, it is unknown whether stem cells behave similarly in the SI and LI, since intravital microscopy has mainly been focused on the SI. Here, we used newly-developed intravital microscopy techniques that facilitate monitoring of clones over multiple weeks in the SI and LI. By combining this analysis with 2D biophysical modelling of the stem cell dynamics, we reveal that the number of effective stem cells in crypts differ for SI and LI, and that this is determined by the degree of Wnt-driven retrograde cell movements.

## Results

### Characteristics of SI and LI crypts

**Extended Data Figure 1:**
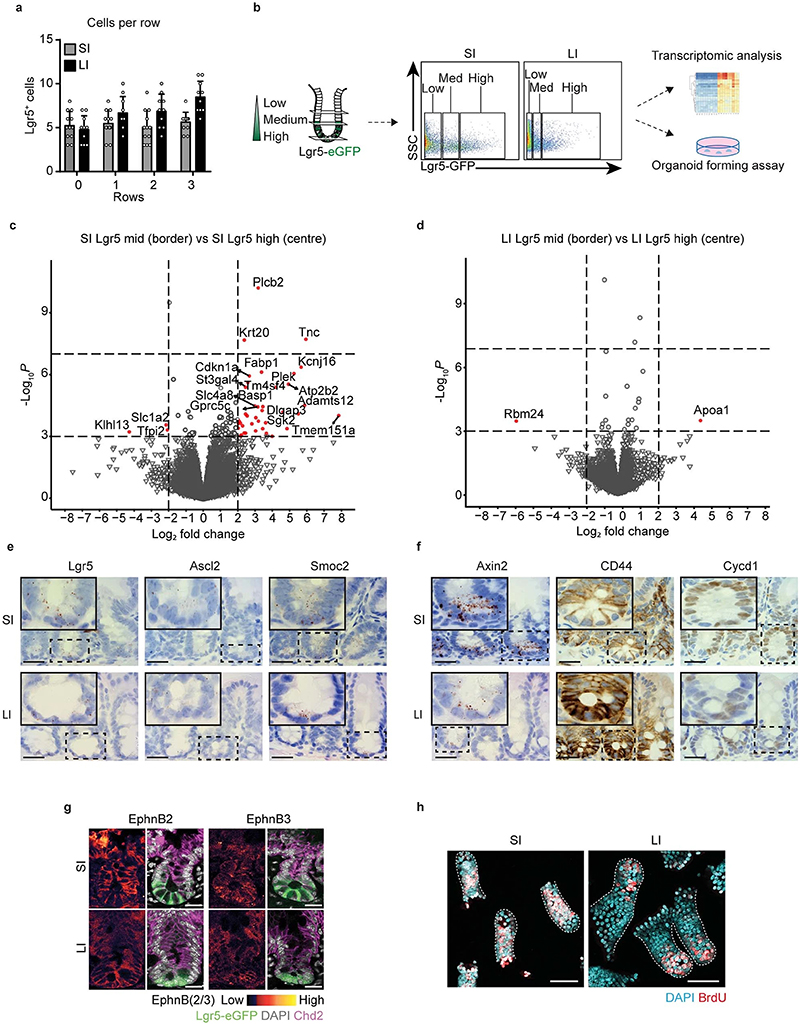
Crypt characteristics in small and large intestine. **a,** Quantification of the number of Lgr5^+^ cells per position in crypt of SI (n=12 crypts) and LI (n=12 crypts) in Lgr5eGFP-Ires-CreERT2 mice. Mean +/- SD are plotted **b**, Schematic representation of experimental setup for RNA-seq and organoid forming assay. **c**,**d**, Volcano plots showing log2 fold-change (x-axis) and -Log_10_ p-value (y-axis) of genes differentially expressed between Lgr5^+^ cells with medium intensity (border) and Lgr5^+^ cells with high intensity (centre). Genes that were significantly altered in border compared with centre Lgr5^+^ cells are highlighted in red (Log_2_ fold change >2, -Log_10_ p-value <0.001) in SI (**c**) and LI (**d**), n=4 mice for each condition. **e**, Stem cell markers *(Lgr5, Ascl2* and *Smoc2*) *in situ* hybridization (ISH) in C57/B6 mouse SI (top) and LI (bottom) crypts, n=4 mice. Scale bar, 100 μm. **f**, Wnt targets (AXIN2, CD44, CYCD1) ISH and immunohistochemistry (IHC) in C57/B6 mouse SI (top) and LI (bottom) crypts, n=4 mice. Scale bar, 100 μm. **g**, Immunofluorescence (IF) staining of Ephrin B2 and Ephrin B3 in C57/B6 mouse SI (top) and LI (bottom) crypts, n=3 experiments. Scale bar, 20μm. **h,** Confocal images of isolated crypts (dotted outline) of SI (left), and LI (right), proliferating cells were identified by BrdU incorporation upon 2-hour pulse (red). Nuclei were labelled using DAPI (blue), n=10 experiments. Scale bar, 50μm.

**Figure 1:**
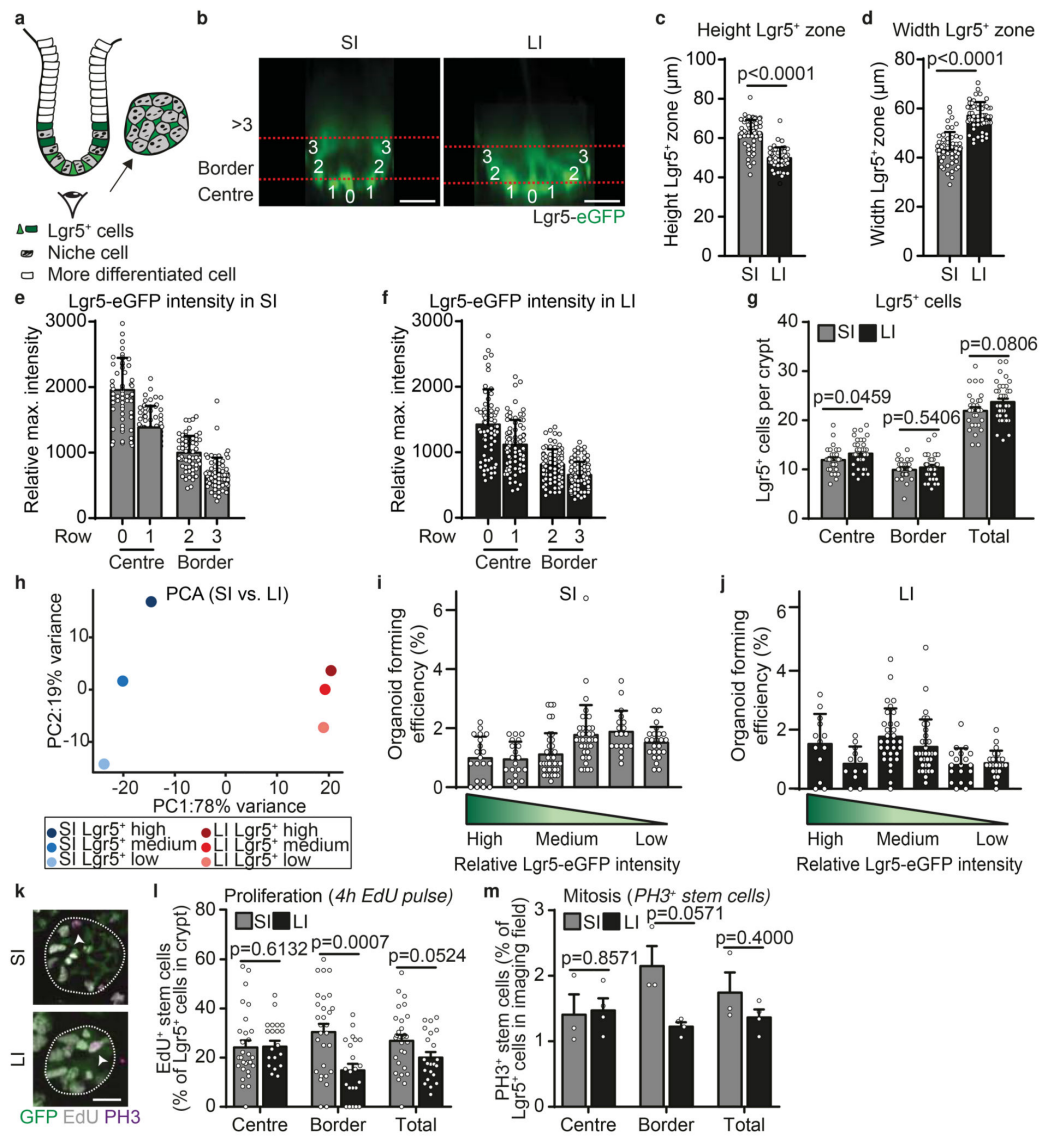
The spatial organization and functional potential of Lgr5+ cells are comparable in the SI and LI. **a,** Schematic crypt representation. **b,** Representative XY-images of SI and LI crypts in Lgr5eGFP-Ires-CreERT2 mice from n=4 experiments. The relative position of Lgr5^+^ cells in the central (row 0 and 1) or border region (row 2 and 3) of the stem cell niche. **c**,**d,** Height (**c**) and width (**d**) of Lgr5-GFP^+^ zone in SI and LI (**c**,**d**, n=50 crypts (SI), n=48 crypts (LI)). **e,f,** Relative maximum intensity of Lgr5-eGFP signal in SI (**e**) and LI (**f**) (**e**, n=47, 62, 61 and 62 crypts; **f**, n=66, 78, 77 and 84 crypts for row 0, 1, 2 and 3 respectively). **g**, Number of Lgr5^+^ cells in centre, border and total in SI and LI crypts (n=50 and 48 crypts, respectively). **h,** Principal component analysis of RNA-seq in Lgr5 high, medium and low cells (i.e. centre, border and >3 row cells). Dots, mean (n=3 mice). **i,j,** Organoid forming efficiency of Lgr5^+^ cells with high, medium and low intensity isolated from SI (**i**) and LI (**j**). Dots, percentage of cells that formed organoids in a BME drop (Left to right, **i**, n= 20, 20, 39, 38, 19 and 24; **j**, n= 13, 12, 32, 34, 19 and 24 BME drops from n=3 experiments in 3 mice). **k**, Confocal images of Lgr5-eGFP cells in S-phase (4h EdU) and mitosis (phospho-histone H3 (PH3), arrow heads) in SI and LI crypts (dotted outline). **l,m,** EdU+ (**l**) and PH3+ (**m**) Lgr5+ cells as a percentage of the total Lgr5^+^ pool in SI (**l**, n=33 crypts; **m**, n=3 image fields) and LI (**l**, n=22 crypts; **m**, n=3 image fields). Bars, mean +/- SEM. Significance in (**c**, **d**, **l** and **m**) was determined by two-sided Mann-Whitney tests. Scale bars: 20μm (**b** and **k**).

To determine which cells act as effective stem cells in the SI and LI, we first looked at the total pool of Lgr5^+^ cells at the crypt bases of Lgr5eGFP-Ires-CreERT2 mice at both sites (Fig. 1a,b). The height of the Lgr5 zone is smaller in the LI than the SI, whilst the diameter of the crypt in the LI is larger than the SI (Fig. 1c,d). In both SI and LI crypts, Lgr5^+^ cells are distributed over ~4 rows, with ~5 cells per row (Extended Data Fig. 1a, Fig. 1b). In both the SI and LI crypts, Lgr5 expression is the highest for cells positioned at rows 0 and 1, and intermediate for cells positioned at rows 2 and 3, defined respectively as centre and border Lgr5^+^ cells (Fig. 1e,f). Cells positioned beyond rows 3 express low levels of Lgr5 (Fig. 1b). In both SI and LI crypts, the centre and border regions contain comparable numbers of Lgr5^+^ cells (Fig. 1g), with a total of approximately 22-24 Lgr5^+^ cells per crypt (Fig. 1g).

To test for potential differences in transcriptional level or colony-forming capacity, we isolated Lgr5^+^ cells from the centre, border and the region beyond the border (row >3) from both SI and LI crypts based on their Lgr5 expression by flow cytometry (Extended Data Fig. 1b). As expected, we observed differences in the transcriptional profile between centre and border Lgr5^+^ cells within SI and LI crypts and more profound differences between the two intestinal sites (Fig.1h, Extended Data Fig. 1c,d). Similar results were found when we performed staining for other (stem cell) markers and Wnt targets *(Ascl2, Smoc2, Axin2, CD44, Cycd1, EphnB2* and *EphnB3)* (Extended Data Fig. 1e-g). Importantly, regardless of any molecular differences, centre and border Lgr5^+^ cells showed similar potential to form organoids in growth-factor rich medium (Fig.1i,j). This data suggest that Lgr5^+^ cells, regardless of their position within the crypt, have the potential to regain the full clonogenic capacity when exposed to factors from the stem cell niche.

In addition to the number of Lgr5^+^ cells and their intrinsic molecular and functional potential, the cell division rate and the position of proliferating cells are important for the dynamics of stem cell competition, since together they determine the rate at which stem cells are replaced in the niche. We compared the presence of proliferating cells in SI and LI crypts by quantifying the number of cells in S-phase (measuring short-term EdU incorporation) and the number of mitotic cells (positive for phospho-histone H3) (Fig. 1k-m). Our analyses revealed similar numbers of proliferating cells in the central crypt regions of the SI and LI (Fig. 1l,m). Moreover, we found that more cells in the border region of SI crypts were proliferating compared to those in the LI (Fig. 1l,m), resulting in a slightly higher fraction of proliferative cells in the SI overall. Of note, the proliferative zone above the Lgr5^+^ zone, known in the SI as the transit-amplifying zone, was significantly less pronounced in the colonic epithelium compared to the SI when assessed by BrdU incorporation (Extended Data Fig. 1h). Together, these results indicate that the spatial organization of Lgr5^+^ cells within crypts and their functional potential are overall comparable between the different intestinal compartments.

### Effective stem cell numbers in SI and LI

**Extended Data Figure 2:**
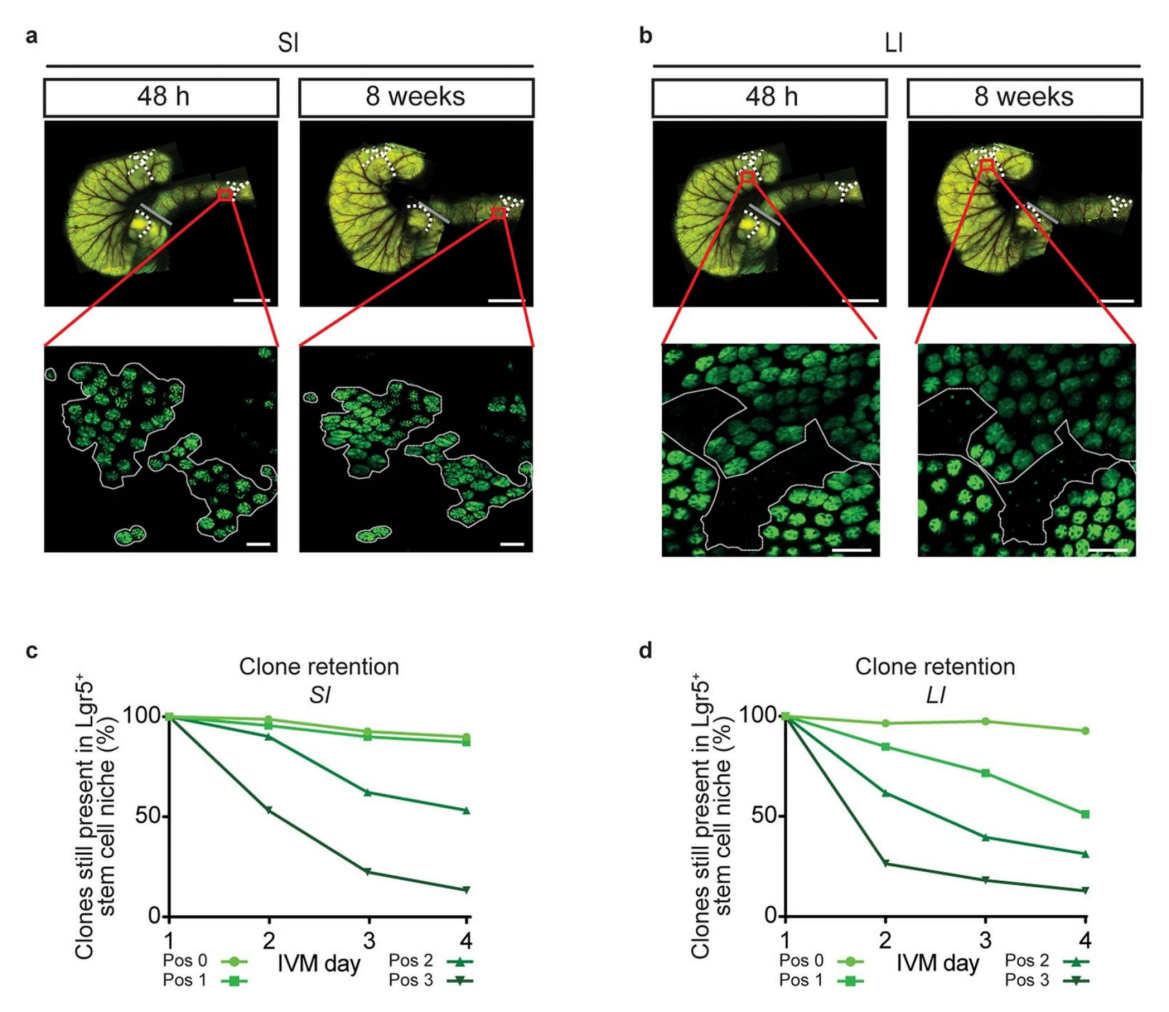
Visualising effective stem cells by intravital imaging in small and large intestine. a,b, Representative overview images 48 hours (left) and 8 weeks (right) after tracing in Lgr5eGFP-Ires-CreERT2 mice from 6 independent experiments. Dotted lines represent same areas. Grey lines indicate SI LI boundary. Lower pictures represent intravital images showing crypt patterns (Lgr5-eGFP in green) at 48h and 8w after tracing in SI (**a**) and LI (**b**). Dotted lines are examples of retraced patchy Lgr5^+^ areas. Scale bar, 5 mm (top), 100 μm (bottom) from 5 independent experiments. **c**,**d**, Quantification of retention within the Lgr5^+^ zone of clones starting from different positions in the niche (shades of green) in SI (**c**) and LI (**d**) as followed by IVM. SI: n=305 clones in 9 mice; LI: n=311 clones in 5 mice (see Ext. Data Fig. 3-6).

**Figure 2:**
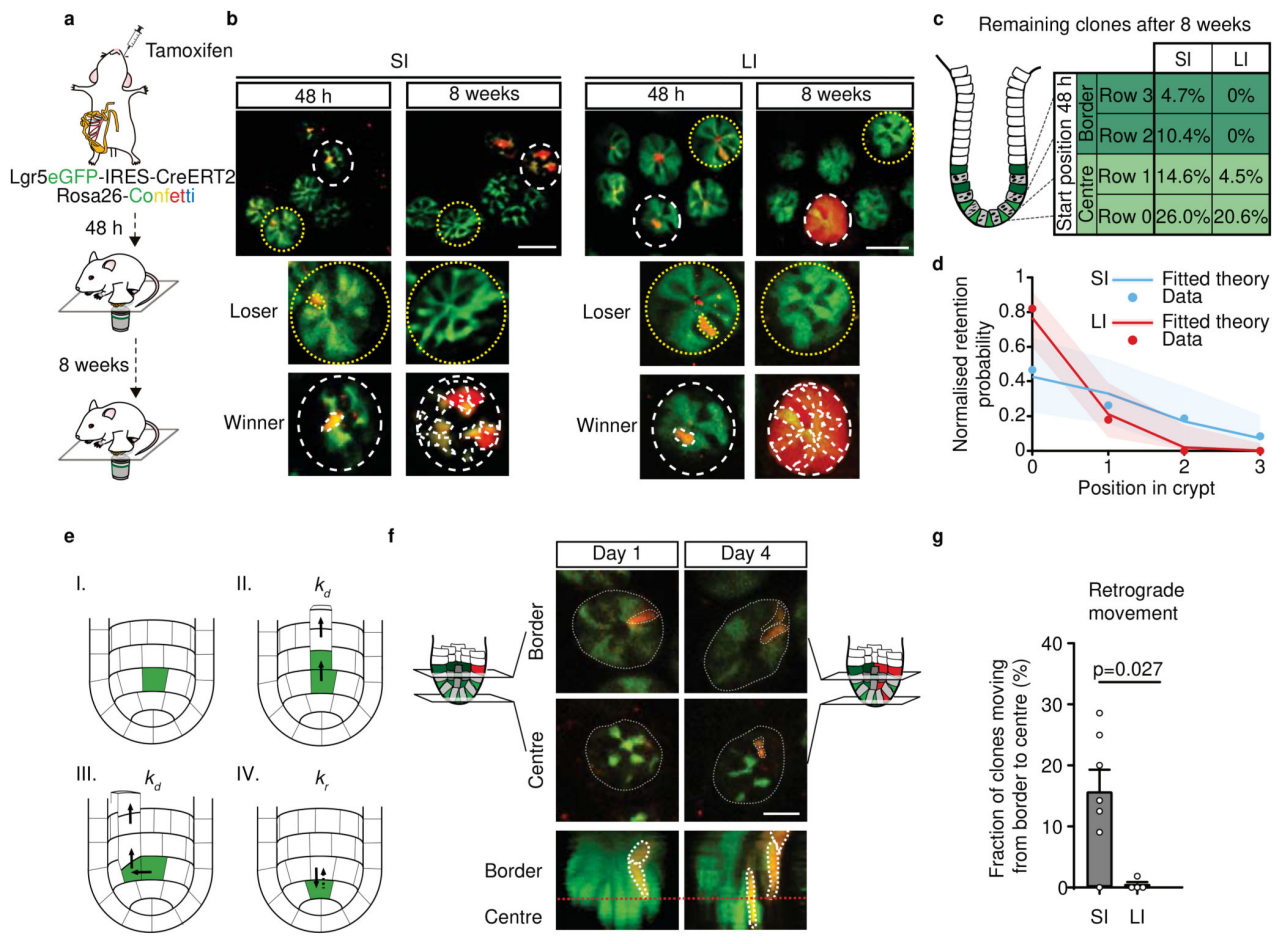
Different numbers of effective stem cells in SI and LI due to retrograde movement. **a,** Schematic representation of experimental setup. **b,** Representative overview images 48 hours and 8 weeks after tracing in SI (left) and LI (right) from 6 independent experiments. Dotted yellow and white circles represent retraced crypts. A labelled clone can either be lost (loser, dashed yellow circle) or retained (winner, dashed white circle). **c,** Clone retention from different starting positions in SI (n=267 clones in 6 mice) and LI (n=294 clones in 6 mice). **d,** Normalised retention probability at 8 weeks as predicted by the model (lines with 95% confidence interval) and experimental data (dots) in SI (n=267 clones in 6 mice) and LI (n=294 clones in 6 mice). **e,** Model sketch: the crypt is abstracted as cylinder coupled to a hemispheric region (I). *k_d_* is the upward movement rate due to cell division (II, III) and *k_r_* is the random cell relocation rate, including retrograde movements (IV). **f**, Example of a border starting clone at day 1 that moved to the centre at day 4 (retrograde movement) from 7 independent experiments. **g,** Percentage of border starting clones present in centre on day 3 in SI (n=59 clones in 7 mice) and LI (n=109 clones in 4 mice). Mean +/- SEM. Significance was determined by a two-sided Mann-Whitney test. Scale bars: 50μm (**b**), 20μm (**f**).

Since the crypt architecture, including the distribution of cells with the same potential to regain the full clonogenic capacity, was found to be similar, we next tested whether SI and LI crypts also contained similar numbers of effective stem cells. For this, we made use of our newly-developed repetitive intravital microscopy techniques that allowed us to trace the progenies of Lgr5^+^ cells at different positions within the crypt over several weeks. The vasculature in large overview images was used as a landmark to find the same region while higher resolution and more zoomed-in images of the patchy expression of GFP and sporadic distribution of confetti-coloured crypts were used to validate the successful tracing of regions of interest (Extended Data Fig. 2a,b). This approach allowed us to retrace the same crypts over multiple imaging sessions and enabled us to study the fate of individual Lgr5^+^ cells over periods of months (Fig. 2a,b).

Using the multiday imaging approach, we recorded the location of clones in crypt bases 48 hour after induction and traced their fate 8 weeks later in Lgr5eGFP-Ires-CreERT2;R26R-Confetti mice (Fig. 2a,b). Cells that gave rise to clones that persisted over the long-term were designated as effective stem cells (Fig. 2b, winner). Quantification of clone retention (i.e., the percentage of clones that remained present in the Lgr5^+^ zone) revealed that centre-derived clones were more likely to persist than border-derived clones, as expected based on previous studies^9^. Strikingly, in the LI, no border-derived clones remained in the stem cell niche 8 weeks after the onset of tracing while, in the SI, ~15% of border-derived clones continued to persist (Fig. 2c). Further dissection of different starting positions within the crypt bases revealed a gradient of positional advantage and clone retention probability, decreasing from centre to border (Fig. 2c,d), which was remarkably steeper in the LI compared to the SI (Fig. 2d). These results indicate that, in contrast to the SI, Lgr5^+^ cells in the border of LI crypts do not function as effective stem cells. Together, we conclude that, during homeostasis, SI crypts contain more effective stem cells than LI crypts.

### Modelling suggests retrograde movements

To better understand why LI border Lgr5^+^ cells do not function as effective stem cells whilst they have the intrinsic molecular and functional potential to do so, we turned to quantitative modelling. Previous studies have sought to model the neutral drift dynamics of clones around the crypt circumference using a minimal one-dimensional scheme^1^ in which the effective stem cell number was either unassigned, linked to Lgr5^+^ expression^2^, or fit using a continuous labelling strategy^10^. However, to understand how this effective stem cell number arises in the first place^11^, we explicitly modelled the two-dimensional organization and biophysical cellular dynamics of the intestinal crypt (see Supplementary Note, sections 1.1 and 2.1). In particular, we modelled individual crypts as regular two-dimensional cylindrical grids (denoting rows 0, 1, etc. as the cell position along the crypt-villus axis, with 5 cells per row, as measured in Extended Data Fig. 1a). In this model, cells undergo two core processes (sketched in Fig. 2e): 1) cell division at rate *k_d_* (with random division orientation), leading to the upward transfer of cells along the crypt-villus axis (i.e., **anterograde movement**); and 2) random cell relocation at constant rate *k_r_*, leading to the exchange of neighbouring cells either within or between adjacent rows, allowing for additional directed onward or **retrograde movement** towards the crypt base. In the absence of retrograde movement (termed the “deterministic conveyor belt” model), cells at row 0 would systematically “win” the competition over cells at rows >0, while non-zero values of *k_r_* (the “stochastic conveyor belt” model) allow for cells at higher rows to participate in the competition, and function as effective stem cells. Crucially, this model makes the generic prediction that the probability of long-term clone retention should decrease with the distance from the bottom of the crypt as a Gaussian-like distribution specifying a well-defined effective stem cell number that depends only on the dimensionless ratio *k_r_/k_d_* (i.e., the ratio of relocation and division rates, Supplementary Note, section 1.2).Thus, this modelling suggests that differences in retrograde movement (i.e., *k_r_*) could provide a mechanistic explanation of the differential fate of the border Lgr5^+^ cells in the SI and LI.

### Imaging retrograde movement intravitally

**Extended Data Figure 3:**
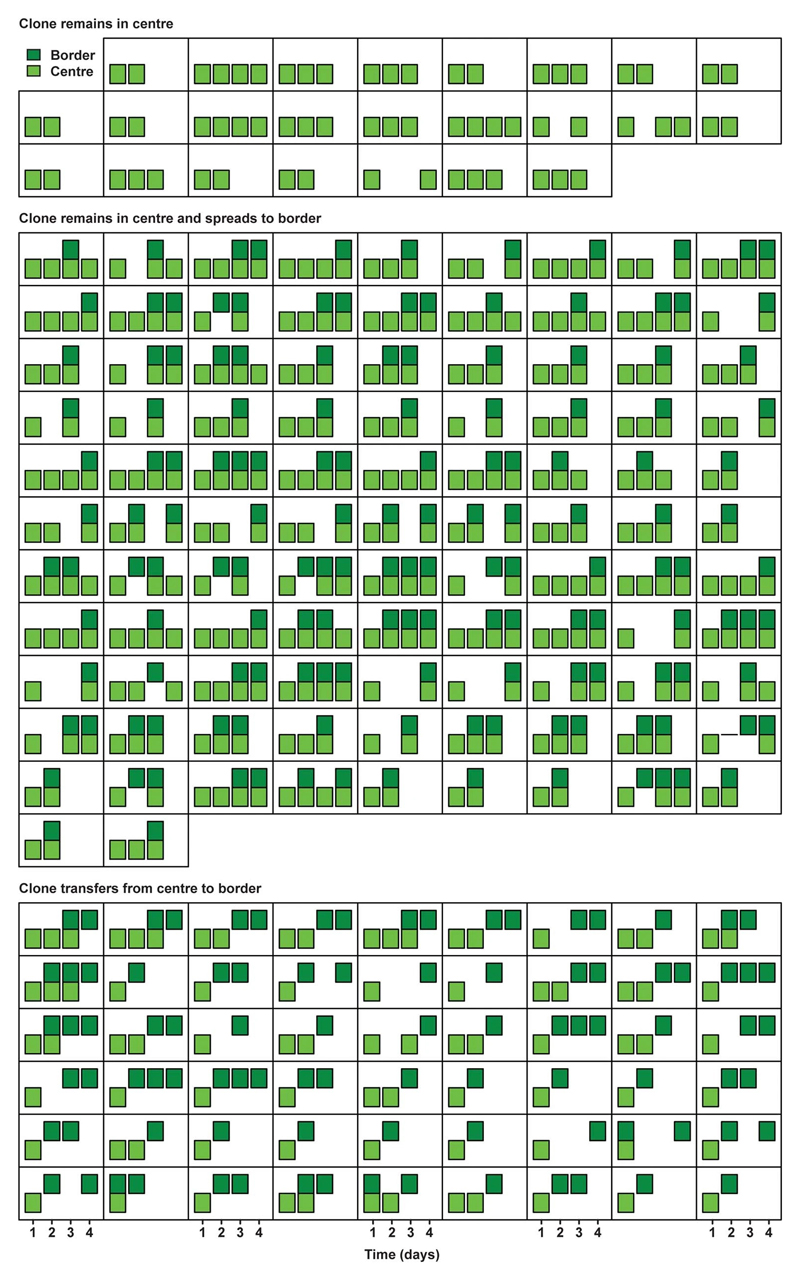
Short-term evolution of clones in SI (1). Presence in centre (light green) and border (dark green) of individual clones in Lgr5eGFP-Ires-CreERT2; R26-Confetti mice followed by short-term IVM in SI is plotted over time (squares represent individual clones, with a bar per day). Plotted are clones starting and remaining in the centre (top panel), starting and remaining in centre while spreading to border (middle panel) and starting in the centre and transferring to border (bottom panel).

**Extended Data Figure 4:**
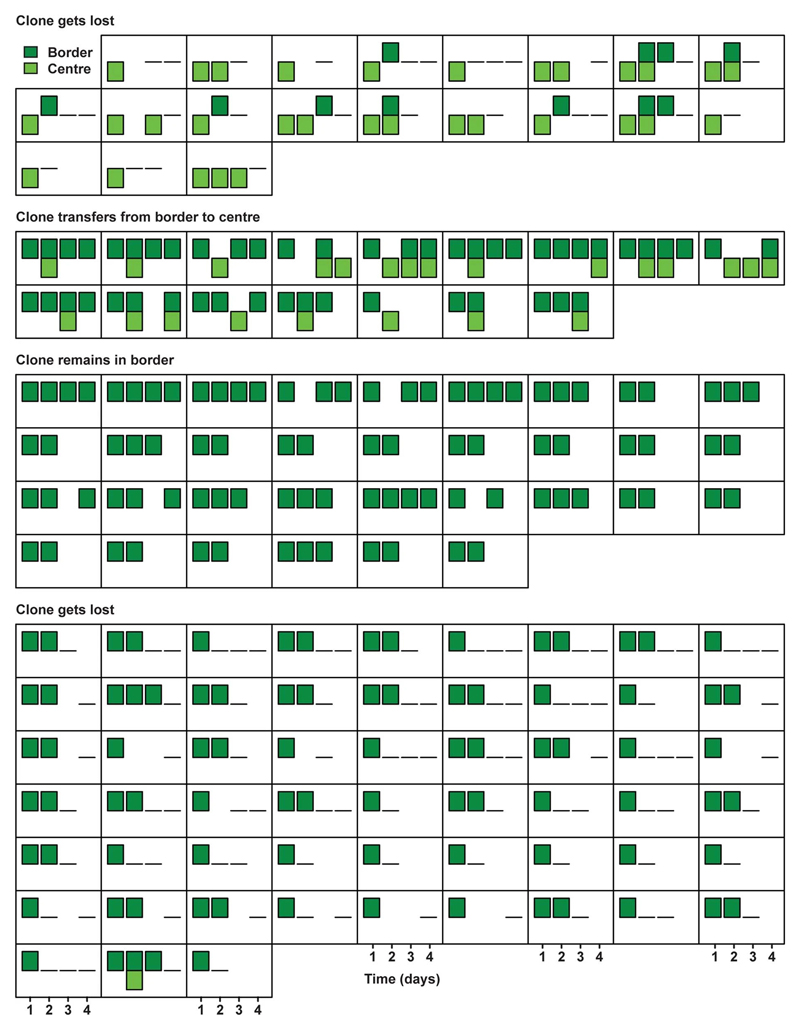
Short-term evolution of clones in SI (2). Presence in centre (light green) and border (dark green) of individual clones in Lgr5eGFP-Ires-CreERT2; R26-Confetti mice followed by short-term IVM in SI are plotted over time (represent individual clones with a bar per day). Plotted are clones starting in the centre and getting lost (top panel), starting in border and transferring to centre (second panel), starting in border and remaining in border, and starting in border before getting lost.

**Extended Data Figure 5:**
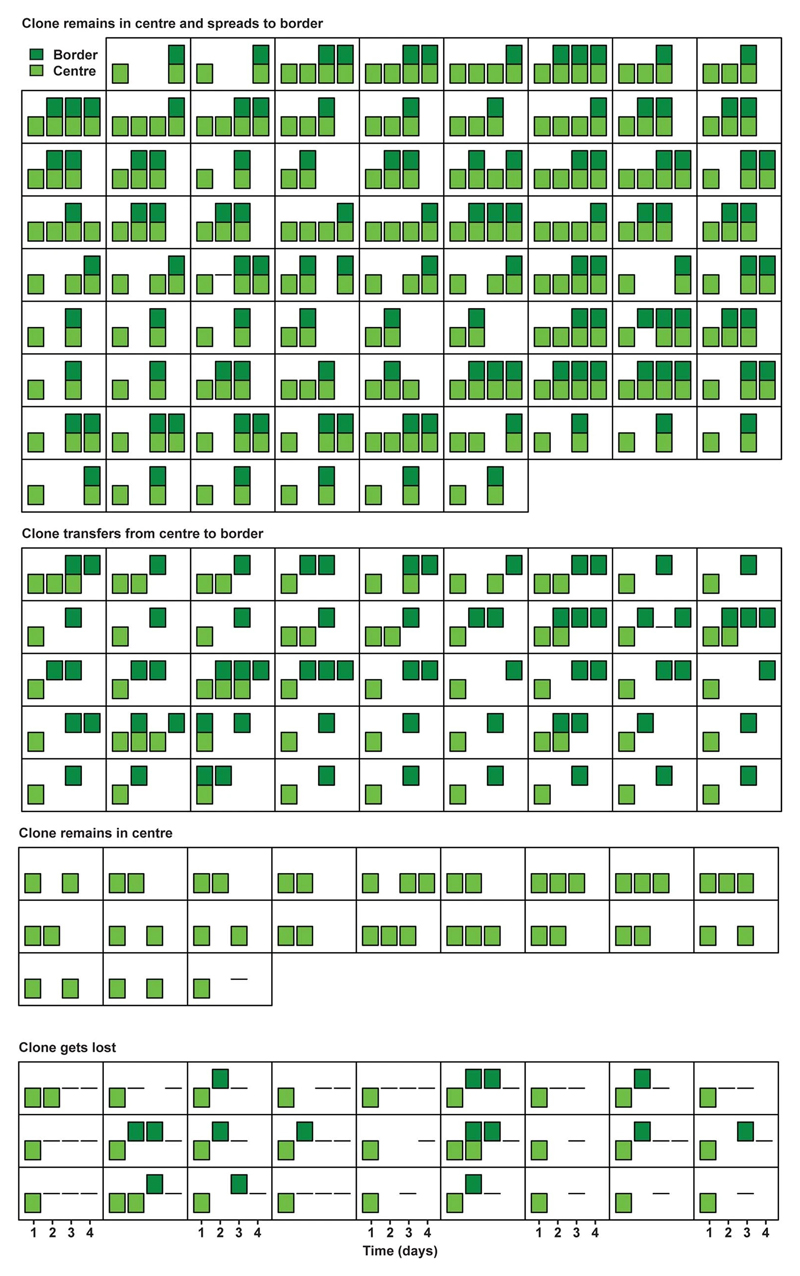
Short-term evolution of clones in LI (1). Presence in centre (light green) and border (dark green) of individual clones in Lgr5eGFP-Ires-CreERT2; R26-Confetti mice followed by short-term IVM in LI are plotted over time (squares represent individual clones with a bar per day). Plotted are clones starting and remaining in the centre while spreading to border (top panel), starting in the centre and transferring to border (second panel), starting and remaining in centre without spreading to border (third panel) and starting in the centre before getting lost from the niche (bottom panel).

**Extended Data Figure 6:**
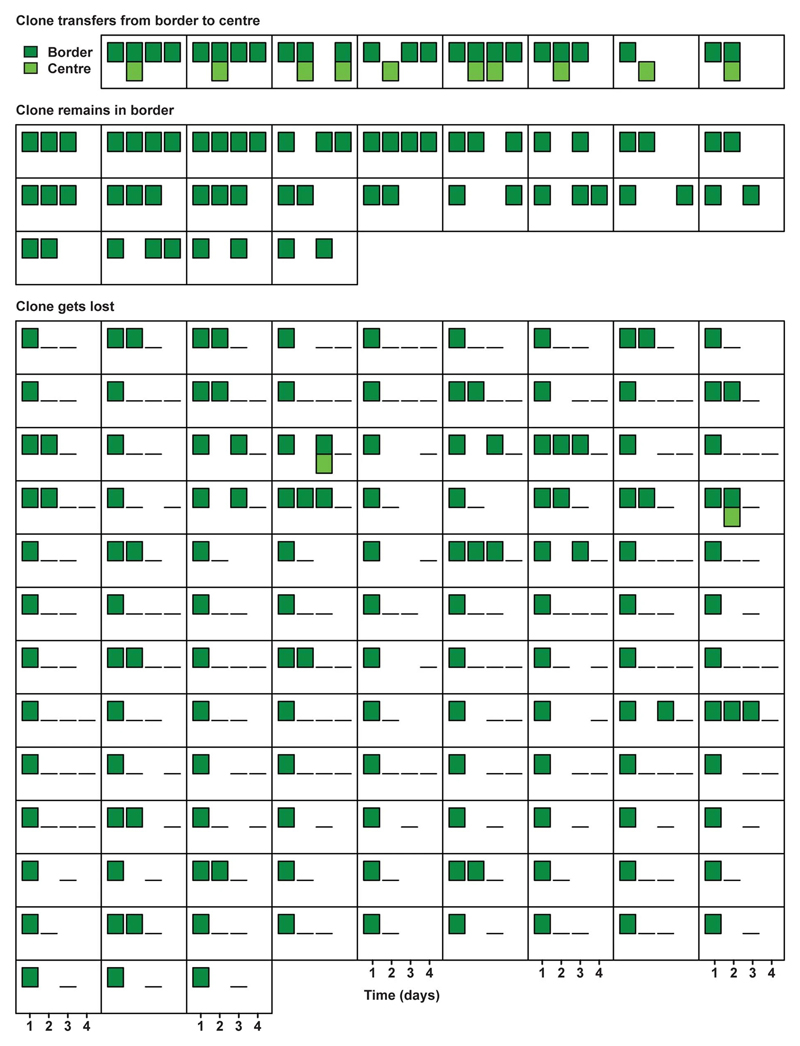
Short-term evolution of clones in LI (2). Presence in centre (light green) and border (dark green) of individual clones in Lgr5eGFP-Ires-CreERT2; R26-Confetti mice followed by short-term IVM in LI are plotted over time (squares represent individual clones with a bar per day). Clones are Plotted are clones starting in border and transferring to centre (top panel), starting in border and remaining there (second panel), starting in border before getting lost (bottom panel).

To test whether the difference of border Lgr5^+^ cells to function as effective stem cells is caused by differential retrograde movement in SI and LI crypts, we performed short-term multi-day intravital microscopy in living Lgr5eGFP-Ires-CreERT2;R26R-Confetti or Lgr5eGFP-Ires-CreERT2;R26-LSL-tdTomato mice. Injection of a low-dose of tamoxifen resulted in Cre-mediated recombination and activation of one of the Confetti colours or tdTomato, respectively – in individual Lgr5-expressing cells. Using intravital microscopy on consecutive days, we monitored cell movement and clonal evolution of cells originally located at central or border regions over time in both the SI and LI (Fig. 2f). Positions and clone persistence were determined and quantified (Fig. 2f and Extended Data Fig. 2c,d and Extended Data Figs. 3-6). As expected qualitatively from the model, retrograde movement of labelled cells was observed in crypts of the SI, but was near-absent in LI crypts (Fig. 2g). In line with the difference in retrograde movement, we confirmed the modelling predictions that clones started from positions away from the crypt base (at levels 2 and 3) in LI crypts are lost faster than their counterparts in SI crypts (Fig. 2c,d, Extended Data Fig. 2c,d).

### Wnt stimulates migration of Lgr5^+^ cells

**Extended Data Figure 7:**
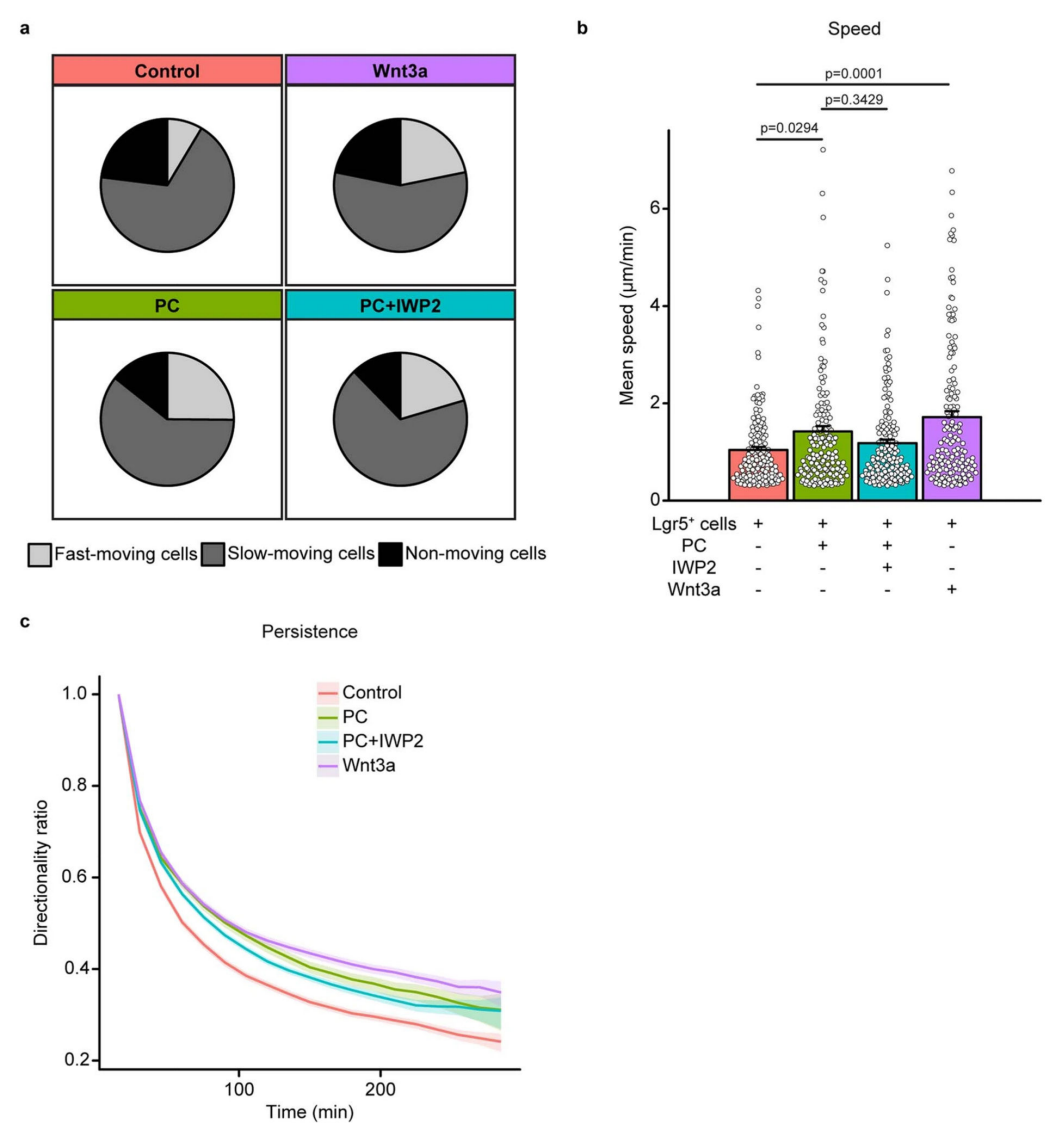
Wnt enhances motility *in vitro.* **a**, Percentage of fast-moving (>2 μm/min), slow-moving (0.3-2 μm/min) and non-moving (<0.3 μm/min) Lgr5^+^ cells. The imaged Lgr5^+^ cells were isolated from Lgr5-EGFP-ires-creERT2;R26R-confetti organoids and exposed to (I) control medium (n=408 cells), (II) medium supplemented with Wnt3a (n=582 cells), (III) medium supplemented with Paneth cells (PC) (n=418 cells), or (IV) medium supplemented with PC and Wnt inhibitor (IWP2) (n=431 cells) in Matrigel from 3 independent biological replicates. **b**,**c** Speed (**b**) and directionality ratio (persistence) over time calculated as mean displacement/length of the trajectory. Significance was determined by a two-sided Mann-Whitney test.(**c**) of single Lgr5^+^ cells in control medium, medium supplemented with Wnt3a, co-culture with PC and co-culture PC with IWP2. Shown are n=150 random cell tracks of Lgr5^+^ cells from 2 independent organoid lines, 50 from each of 3 independent biological replicates. Each point represents the mean value of each track. Shown are mean ± SEM.

**Figure 3:**
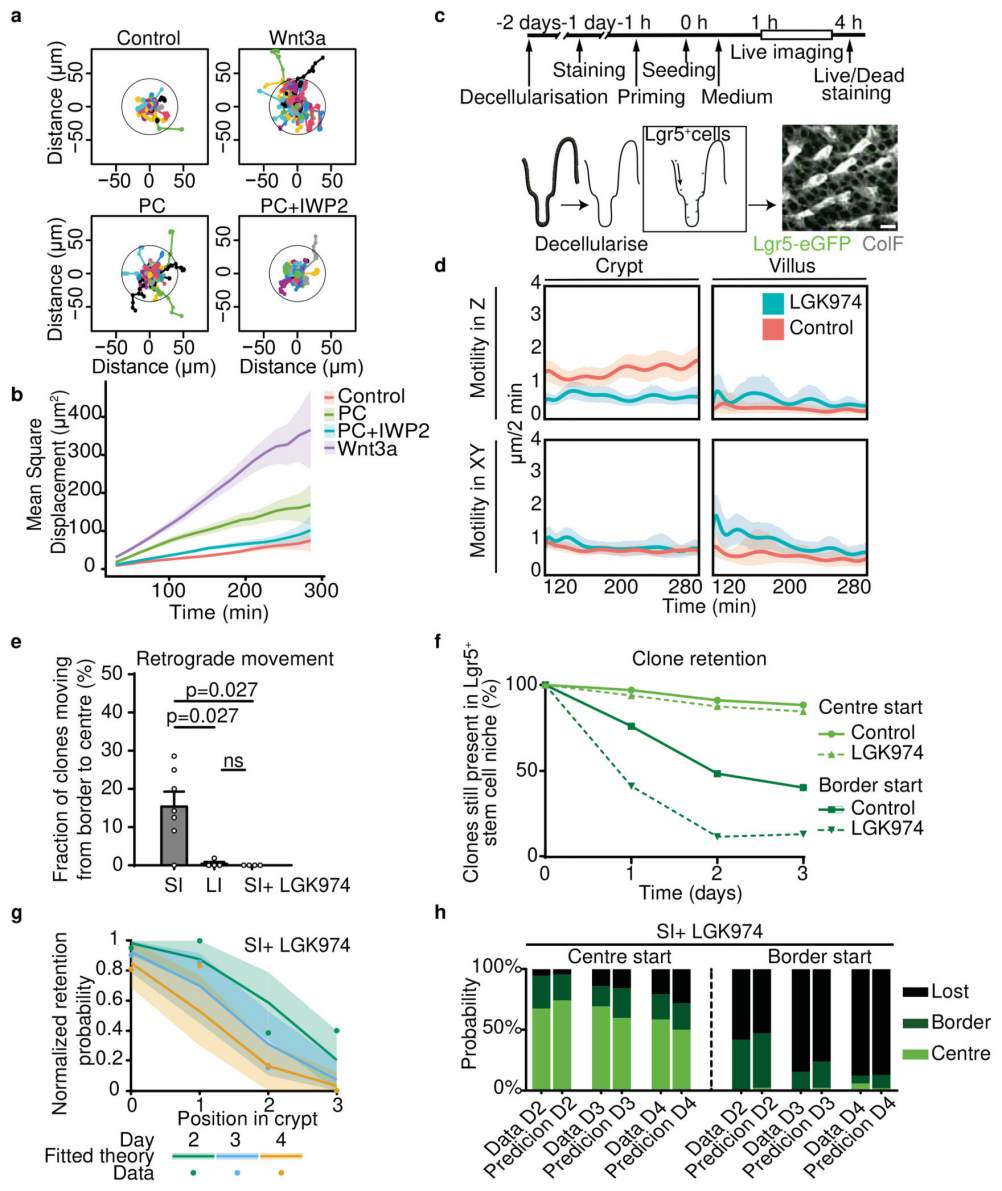
Wnt promotes Lgr5^+^ cell migration. **a**, Normalised migration tracks of single Lgr5^+^ cells isolated from Lgr5-EGFP-ires-creERT2;R26R-confetti organoids in control medium, medium supplemented with Wnt3a, co-culture with Paneth cells (PC), or co-culture PC with Wnt inhibitor (IWP2) in Matrigel (n=150 random tracks of Lgr5^+^ cells from 2 organoid lines, 3 biological replicates). **b,** Mean square displacement (MSD) calculated as a function of time (n=150 cells, 3 biological replicates). Data, mean +/- SEM. **c**, Schematic representation of experimental setup for analysing cell movement on decellularised intestinal scaffolds. **d**, The motility of Lgr5^+^ cells was determined along the crypt-villus axis (motility in Z) and along the lateral axis (motility in XY) (n=150-200 cell tracks from 3 decellularised intestines for each control (vehicle) and LGK974 treated group). Data, mean with 95% confidence interval. **e**, Percentage of border starting clones present in the crypt centre on day 3 (n=59, 109, 75 clones in 7, 4, 4 mice for SI, LI and SI upon LGK974 treatment respectively). Bars, mean +/- SEM. Significance was determined by a two-sided Mann-Whitney test. **f**, Quantification of retention within the Lgr5^+^ zone of centre- or border-starting clones in crypts of control (solid lines) and LGK974-treated (dashed lines) mice as followed by IVM (n=75 clones in 4 mice). **g,** Normalised retention probability in the Lgr5+ zone in LGK974-treated SI (n=75 clones in 4 mice, data re-analysed from^16^ predicted by model (solid lines, mean with 95% confidence interval) and experimental data (dots). **h**, Probability of a clone starting either in niche centre (left) or border (right) to be present in the centre, border or to be lost from the Lgr5^+^ zone over time in SI of LGK974-treated mice, comparing data (left bar) and theory (right bar) (n=75 clones in 4 mice). See Supplementary Note, section 2.2.3 for details. Scale bars: 50μm (**c**).

Next, we aimed to determine the factors that drive retrograde cell movement. Recently, Wnt signalling has been shown to induce migration of intestinal stem cells in drosophila^12^. Wnt is implicated as one of the major niche factors to induce stem cell potential, and is produced by **Paneth cells** (**PCs**) and pericryptal stromal cells in SI crypts^13^. By contrast, LI crypts do not harbour PCs^13^. To test whether (PC-produced) Wnt has the potential to induce migration of Lgr5^+^ cells, we isolated Lgr5^+^ cells by flow cytometry. Next, we seeded these cells on ultra-low attachment round-bottomed plates and imaged their migration with confocal microscopy, as previously described^14^ (Supplementary Video 1). Interestingly, we observed that the mean square displacement of migratory cells was enhanced when cells were exposed to PC (Fig. 3a,b), an effect caused by both enhanced speed and directional persistence (Extended Data Fig 7). Importantly, this migration enhancement was abolished when the secretion of Wnt was inhibited by a **porcupine inhibitor** (**IWP2**) (Fig. 3a,b). These results suggest that PC-secreted Wnt can promote the migration of Lgr5^+^ cells. Indeed, when we directly stimulated Lgr5^+^ cells with exogenous Wnt, migration was enhanced even further (Fig. 3a,b, Extended Data Fig 7).

To further validate the induction of retrograde movement by Wnt, we decellularised the intestines from control and porcupine inhibitor-treated mice (Fig. 3c). Such decellularised extracellular matrix (dECM) scaffolds retain the original intestinal tissue architecture and multiple ECM bound factors that can provide positional cues guiding cell movement *in vivo*^15^ (Fig. 3c). Next, we seeded single Lgr5^+^ cells on top of these scaffolds and observed by live microscopy that individual cells displayed highest motility along the Z axis in crypts, an effect which was absent in villi (Fig. 3d, Supplementary Video 2). Moreover, cell motility along the Z axis was decreased specifically in the crypts of those scaffolds that were prepared from mice treated with the porcupine inhibitor LGK974 (Fig. 3d).

**Extended Data Figure 8:**
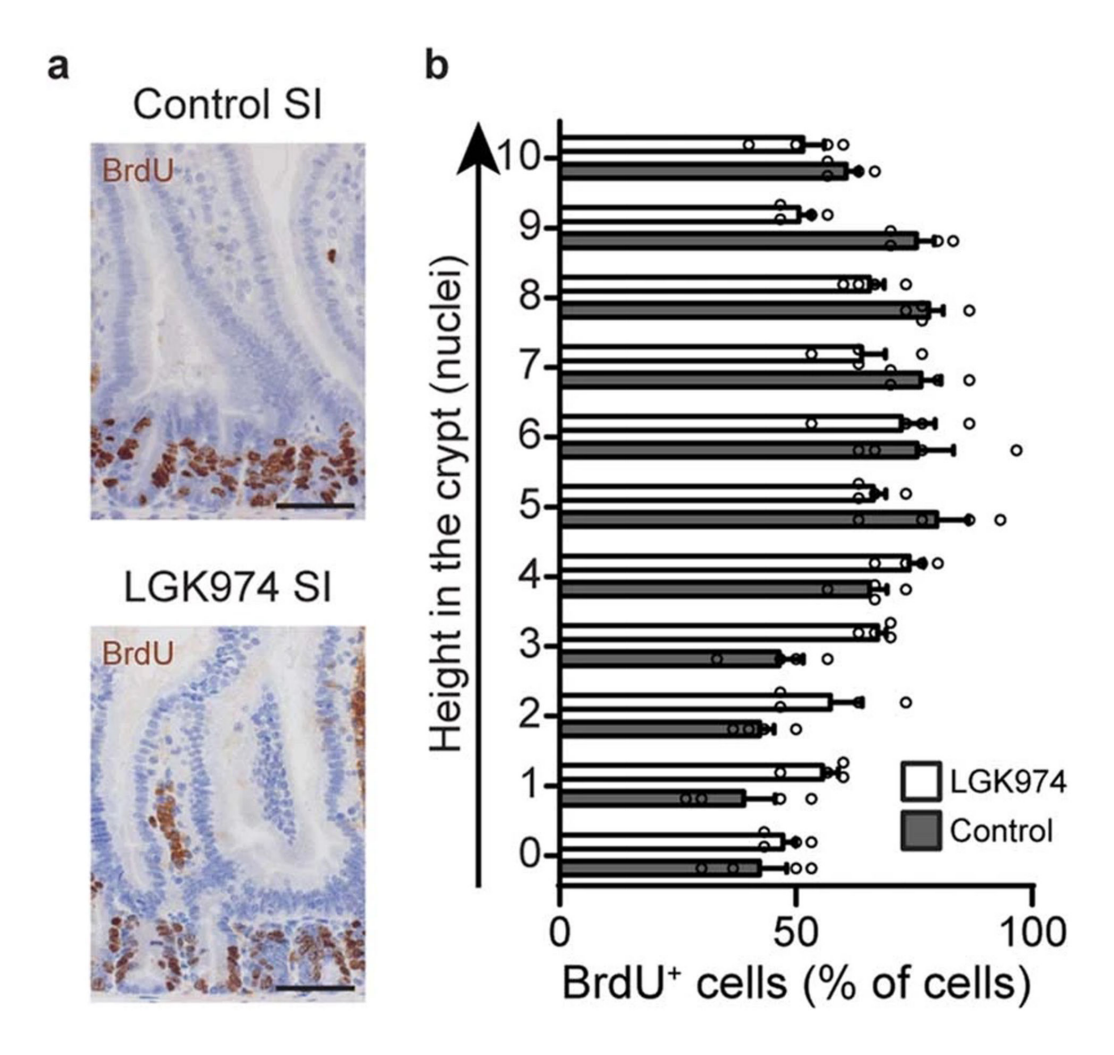
The effect of LGK974 on stem cell dynamics in small intestinal crypts. **a,** Representative image of 2h BrdU pulse in SI crypts of control and LGK974-treated mice. Scale bar, 50μm. **b,** Quantification of cells positive for BrdU per position in SI crypts of control and LGK974-treated mice. Of note, position is based on nuclei count which does not discriminate between stem cells and PCs, and the Lgr5+ zone ends around nuclear position 6-8. Mean +/- SEM are plotted. (n=120 crypts examined over 4 independent experiments from 4 mice, 30 crypts per mouse).

Lastly, we tested whether inhibition of Wnt release also blocks the retrograde movement that we observe *in vivo*. Previously, we have shown that reduced Wnt secretion upon treatment with low dose LGK974 results in a lower number of effective stem cells^16^. By re-analysing^16^ our intravital microscopy data, we indeed found that this change was correlated with a near-abolition of retrograde cell movement (Fig. 3e) as well as decreased clone retention (Fig. 3f), while the cell proliferation rate was hardly affected by LGK974 treatment (Extended Data Fig. 8a,b). When combined, in addition to its classical role as a promoter of self-renewal in the base of crypts, our data suggest that Wnt stimulates cell migration and mediates retrograde cell movement.

### Retrograde movement affects stemness

Our modelling and experiments suggest that Wnt-dependent retrograde movements enable border Lgr5^+^ cells to function as stem cells (i.e., to function as an effective stem cell). However, the extent of this effect and the long-term consequence of differences in retrograde movement (i.e., *kr)* on effective stem cell number in SI and LI crypts remained to be investigated quantitatively. The predicted Gaussian dependency of the probability of clone persistence as a function of initial position along the crypt-villus axis allowed us to define the number of effective stem cells in crypts (Fig. 2d), as the number of cells whose lineage has a significant probability to persist over the long-term (set above 5% for a given row, comprising *2σ* of the fluctuations, where σ^2^ ~ *k_r_/k_d_*, see Supplementary Note, section 1.2).

Consistent with this simple prediction, which did not depend on the decided implementation of retrograde movements in the model (Supplementary Note, section 1.4), the clonal persistence at 8 weeks post-induction as a function of starting position was well-fitted by a Gaussian function in both the SI and LI. From this fit (Fig. 2d and Supplementary Note section 2.2 for details), we obtained: *k_r_/k_d_=0.25* in the LI (95% CI: 0.05-0.55) and *k_r_/k_d_=2* in the SI (95% CI: 0.5-4.5).

Next, we estimated the consequence of differential *k_r_* on the number of effective stem cells, *N_s_* using the predicted relation (see Supplementary Note section 1.3 for details):

$$N_{s}\approx N_{g}(1+2\sqrt{\frac{k_{r}}{k_{d}}})$$

where *Ng* = 5 is the number of cells per row (Extended Data Fig 1a). This led to a figure of *Ns^SI^~19* effective stem cells per crypt in the SI (accounting for nearly all Lgr5^+^ cells) and only around *Ns^LI^*~10 effective stem cells per crypt (translating to the first two rows) in the LI.

**Extended Data Figure 9:**
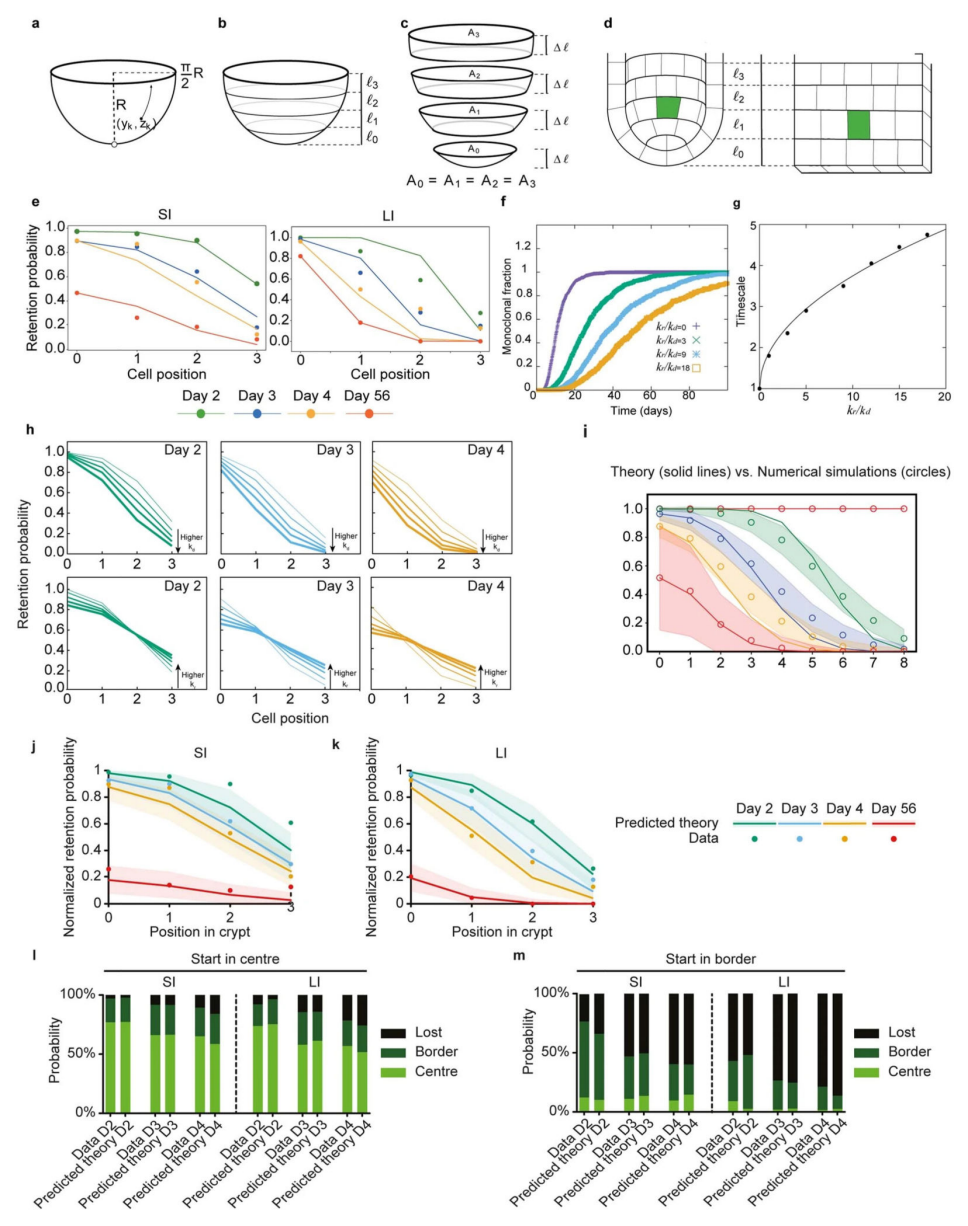
Biophysical modelling of stochastic conveyor belt dynamics in small versus large intestine. **a,** An intestinal crypt is abstracted as a hemispherical surface. A cell experiences net upwards force due to the divisions taking place at lower positions, together with stochastic repositioning events. **b,** This hemispheric region can be segmented by cuts at different heights, ℓ0, ℓ1, ℓ2, ℓ3. **c**, If the sections defined by these cuts are of the same width, Δℓ, then the area of each is the same, which provides an explanation for the near-constant number of Lgr5+ cell at each position. **d**, This allows us to approximate the system as consecutive layers of cells on a cylinder. **e**, Analytical solutions for the stochastic conveyor belt model (probability of clone retention per time). Left (resp. right) plot shows the retention probability as a function of the starting position of the mother cell of the lineage for the SI (resp. LI). Points show the experimental data for wild-type (same as Fig. 3), lines are the prediction of the stochastic conveyer belt dynamics given by equation (1.3) of the SI Theory Note. In both panels, the color scheme is: Green, 2 days, Blue, 3 days, orange, 4 days, and red 56 days post-labelling. **f**, Average monoclonal conversion in crypts for different values of *kr kd* and rescaled time it takes to convert. **g**, Corresponding time of conversion as a function of *kr kd* (points) which are very well fitted by a square root (lines), showing that the time increases close to linearly with *✓kr /kd.* **h**, Sensitivity analysis of the 2D numerical simulations. Top, effect of increasing values of the division rate kd on the resulting short-term clonal retention dynamics as a function of initial cell positions at days 2, 3 and 4 (left, middle and right panel respectively), for constant k_r_=0.25 (LI best-fit value). Increasing thickness of the lines indicate increasing division rate (or alternatively decreasing division time: 2.3, 1.7, 1.4, 1.2, 0.9 divisions per day respectively - note that the middle curve thus corresponds to the value of 1.4 divisions per day used in the main text). Bottom, Effect of increasing values of the division rate k_r_ on the resulting short-term clonal retention dynamics as a function of initial cell positions at days 2, 3 and 4 (left, middle and right panel respectively), for constant k_d_=0.5 (LI best-fit value). Increasing thickness of the lines indicate increasing k_r_=0.25,1,2,3, 4 (note that the first curve thus corresponds to the best fit value used in the main text). **i**, Comparison between 1D analytical theory (solid lines) and 2D simulations (circles) for the clonal retention probability (y-axis, parameters chosen as kr=2, 1/kd=1.2 divisions per day) as a function of initial starting position for the clone (x-axis) and time (colors red, green, blue, yellow and red indicating resp. day 1, day 2, day 3, day 4 and day 56). Dashed region indicates the standard deviation observed in the simulations for the respective simulation time. **j,k,** Normalised probability of retention in Lgr5+ zone for different starting positions over time in SI (f; n=305 clones in 9 mice) and LI (g; n=334 clones in 5 mice) predicted by model (solid lines and shaded intervals, mean with 95% confidence interval) and experimental data (dots). **l,m,** Probability of presence in centre, border or loss of centre-starting (left) and border-starting clones (right) over time in SI (h, n=305 clones in 9 mice) and LI (I, n=311 clones in 5 mice), comparing data (left bar) and theory (right bar).

To validate experimentally the consequence of retrograde cell movement on the number of effective stem cells, we compared the evolution of clone retention as a function of starting position to predictions from numerical simulation and analytical theory, using the same values of *k_r_/k_d_* inferred above. For the short-term dynamics, the proliferation rate *k_d_* also enters in the prediction, setting the overall time scale of the dynamics, with its inferred value broadly consistent with EdU data despite some variations between best-fit value between conditions, which could be related to short-term biases during lineage-tracing (see Supplementary Note section 1 and Extended Data Fig. 9a-g for details). These results provided a good quantitative agreement at every time point for both the SI and LI (Extended Data Fig. 9h,i). In addition, the model and data showed an excellent quantitative agreement (see Supplementary Note section 2 for statistics) for the retrograde movement of clones, indicating that (differences in) both short- and long-term dynamics in the SI and LI can be captured by our model (Extended Data Fig. 9j-m). Thus, the analysis of the short-term dynamics corroborates the findings of long-term lineage data and the values of *k_r_/k_d_* extracted above, confirming experimentally that the retrograde movement determines the effective stem cell number in SI and LI crypts.

Next, we perturbed retrograde movement by inhibiting Wnt signalling using the porcupine inhibitor LGK974. Fitting the model to the dynamics of clone retention as a function of starting position confirmed that LGK974 treatment decreased *k_r_/k_d_* to 0.4 (95% CI: 0.-1), resembling the value found in the LI during homeostasis. As well as providing a good fit of the clone retention dynamics (Fig. 3g), this ratio also predicted the rate of movement of clones between centre and border compartments (Fig. 3h, see Supplementary Note section 2.2 for details).

**Extended Data Figure 10:**
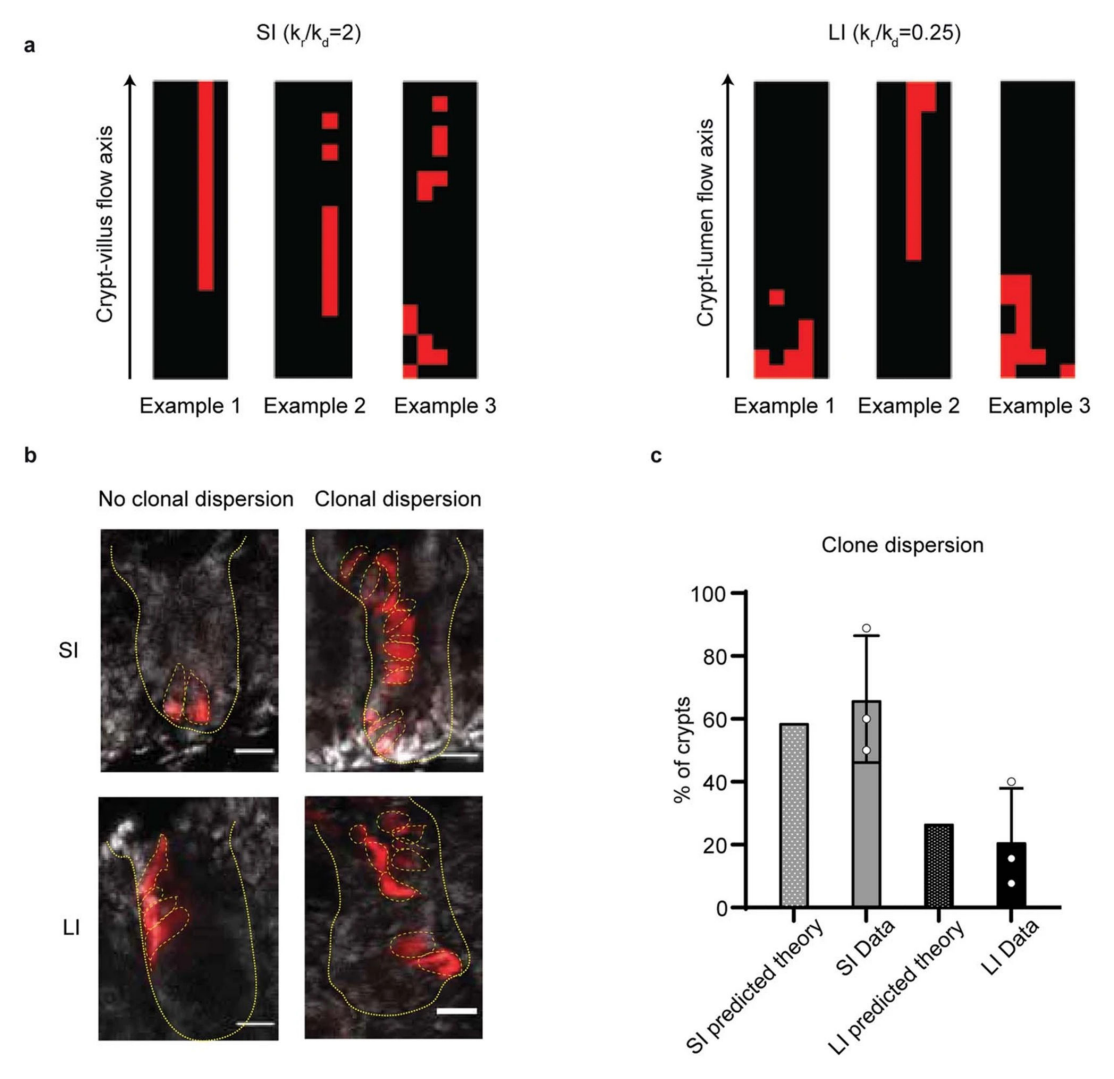
Clonal dispersion in small and large intestine. **a**, Typical outputs of 2D numerical simulations of a single clonal labelling event (labelled cells indicated in red) for the parameter set extracted from SI (left) and LI (right) data. As expected, larger values of kr result in a higher probability of clonal fragmentation (defined as the probability of a given clone displaying two fragments separated by a row of clonally non-labelled cells, see SI Note for details on the simulations). **b**, SI (top) and LI (bottom) crypts with sparse lineage-tracing experiment, where a single lineage (red here, induced and imaged 7 days post induction) can be observed. Clonal dispersion due to cell rearrangements is either observed (right) or not (left). Scale bar, 20μm. **c**, Probability of clonal fragmentation in SI and LI (data shown in grey (SI) and black (LI), theory in dotted bars extracted from the parameters in panel a), showing good agreement. Data is based on n=3 mice (20 crypts for SI and 55 crypts for LI). Each data point represents percentage of clonal dispersed crypts in one mouse, and bars show mean +/- SD.

Finally, we reasoned that a large retrograde movement rate *k_r_* in the SI should also have a consequence for the spatial dispersion of clones along the crypt-villus axis. To test this, we performed short-term tracing, where we reconstructed clones along the crypt-villus axis, both in the centre-border and beyond border compartments. Importantly, we found that the probability of clone fragmentation was much higher in the SI than the LI (Extended Data Fig. 10), and that the magnitude of the difference was well-predicted by our 2D spatial model with the same parameters *k_r_/k_d_* extracted above, providing an independent confirmation for the predictions of the model. Together, these experiments further strengthen our conclusions that different short-term retrograde movement can explain differences in the long-term retention probability of crypt cells, and thereby dictate different effective stem cell numbers in SI and LI crypts.

### Retrograde movement alters monoclonality

**Figure 4:**
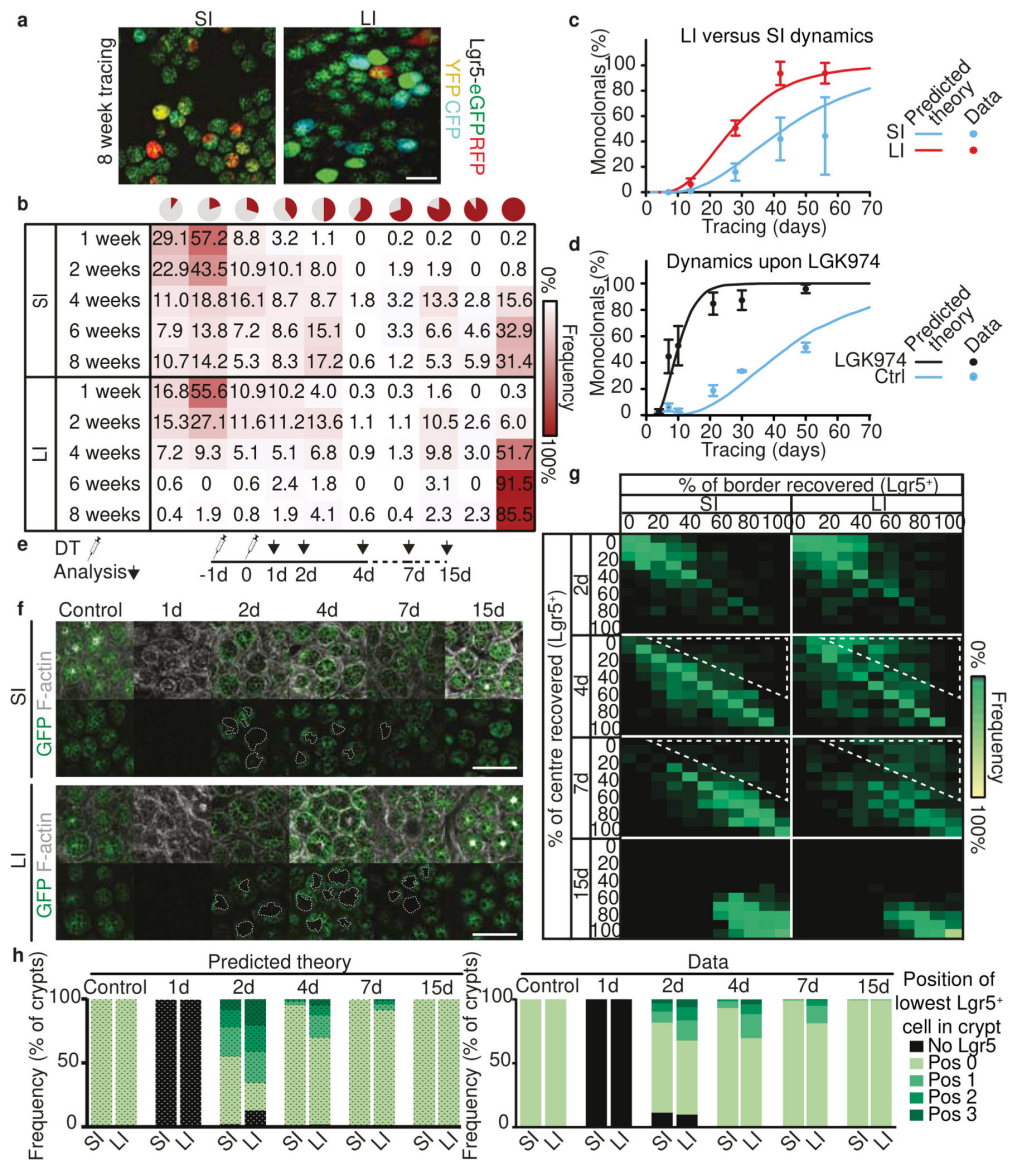
Consequences of retrograde movement. **a,** Representative maximum projections of crypt bottoms 8 weeks after induction in SI and LI (n=5 independent experiments). **b**, Heat map showing frequency of clone sizes at different time points in SI and LI. **c**, Monoclonal crypts in SI and LI over time predicted by model (solid line) and real data in SI and LI. Dots, mean +/- SD (**b**,**c**, n= 444, 375, 218, 152, 151 clones in 3, 4, 3, 5, 7 mice (SI); n= 304, 465, 236, 164, 531 in 3, 4, 3, 5, 8 mice (LI) for 1, 2, 4, 6 and 8 weeks respectively). **d**, Monoclonal crypts in SI of control and LGK974 treated mice (n for control= 3, 3, 4, 4, 3, 3 mice; n for LGK974= 2, 4, 3, 5, 3, 3 mice for day 4, 7, 10, 21, 30, and 50 respectively, 200 clones per mouse) over time predicted by model (solid line) and real data reanalysed from^16^. Dots, mean +/- SEM. See Supplementary Note section 2.2.3 for details. **e**, Schematic representation. Targeted ablation of Lgr5+ cells by two DT injections in 19 Lgr5DTR:EGFP mice. **f**, Representative images of untreated and DT treated mice at different time points post ablation (n= 3 independent experiments). **g**, Heatmap of the percentage of crypts recovered in centre and border regions in the SI and LI (n= 5, 3, 4, 3 mice for day 2, 4, 7, and 15 respectively). Note the faster recovery kinetics of SI crypts (white highlighted area). **h**, Best numerical fit for the position of lowest Lgr5^+^ cell using the biophysical model (left), predicting a faster dynamics in the presence of a larger amount of retrograde movements, and experimental data (right) (n= 2, 3, 5, 3, 4, 3 mice for control, day 1, 2, 4, 7, and 15 respectively). Scale bars: 200μm (**a**), 100μm (**f**).

To examine the consequence of different effective stem cell numbers in SI and LI crypts, despite showing similar crypt characteristics and near-equal number of Lgr5^+^ cells, we next compared the time it takes for one clone to outcompete all others in a given crypt, i.e., the time that it takes to reach crypt monoclonality. For this, we used whole mount preparations of Lgr5eGFP-Ires-CreERT2;R26R-Confetti mice sacrificed at different time points after onset of lineage tracing (Fig. 4a). As predicted by our model, we observed differences in clonal expansion over time within the Lgr5^+^ zone of SI and LI crypts, with faster evolution (i.e. growth) of LI clones compared to SI clones (Fig. 4b). Monoclonality was reached faster in the LI than in the SI: whereas only ~30% of SI crypts were monoclonal at 6 weeks after onset of tracing, the vast majority of LI crypts was already monoclonal at that time (Fig. 4a,b). Importantly, this evolution was predicted quantitatively by our model (Fig. 4c) with the different estimated ratios of *k_r_/k_d_* for each region (while the geometry, i.e., number of cells competing neutrally in a given row, and average cell division rate, were equal). Moreover, our model prediction showed good agreement with the experimental long-term clonal evolution data upon Porcupine inhibitor treatment (Fig. 4d and Supplementary Note, section 2.4). Together, this argues that the process of monoclonal drift in intestinal crypts is faster in LI than SI, and can be enhanced by lowering the number of effective stem cells due to reduced retrograde movement upon Porcupine inhibition.

### Retrograde movement impacts regeneration

Movement of cells from the transit-amplifying zone into the stem cell zone has been shown to be important for the regeneration of the stem cell pool following their loss^17^. Therefore, to test whether distinct retrograde movement in SI and LI crypts also affects regeneration of the stem cell pool, we traced the recovery of the SI and LI Lgr5^+^ cell compartment following targeted ablation of Lgr5^+^ cells using diphtheria toxin (DT) injection in mice where the human DT receptor (DTR) fused to EGFP was knocked into the Lgr5 locus (Lgr5DTR:EGFP)^17^. We measured the recovery of Lgr5-DTR-GFP cells at various cell positions in the crypt (Fig. 4e-g). We first performed this experiment *in silico* using our mathematical framework (Fig. 4h, left). Due to differences in the retrograde movement, our framework predicted that the recovery of the stem cell pool in the SI should be faster than in the LI, especially regarding the stem cells in the centre of the niche (position 0 and 1). Strikingly, we indeed observed such distinct behaviour in experiment (Fig. 4h, right). Whilst the recovery of centre Lgr5^+^ cells was near complete after 4 days in the SI, a large proportion of the LI crypts still lacked a centre Lgr5^+^ cell after 7 days (Fig. 4h, right and indicated in the white dotted area in Fig. 4g). Combined, this data suggests that the retrograde movement supports fast regeneration of the Lgr5^+^ cell pool.

## Discussion

Stem cells are defined by their potential to self-renew and give rise to differentiated progeny. Traditionally, stem cell potential has been thought to be an intrinsic property induced by cues such as Wnt, and characterised by signature expression of molecular markers such as *Lgr5.* Whilst Lgr5^+^ cells in intestinal crypts have the intrinsic capacity to give rise to all differentiated cell types and to form *in vitro* organoid cultures, Lgr5 expression does not necessarily overlap with the ensemble of cells showing functional stem cell activity^4–8^. Here we have shown that, in addition to the passive rearrangements of cells upon proliferation (i.e., cells that are displaced by others), Wnt-driven active retrograde movements (i.e., cells that move by themselves in the opposite direction of anterograde movement) determine the number of effective stem cells in intestinal crypts. Although Lgr5^+^ cells at the border of the LI crypt base (rows 2 and 3) are exposed to stem cell-inducing niche and have all the properties to function as stem cells, we find that, due to lack of retrograde movement, these cells are not able to form long-term clones and to act as effective stem cells as they get continuously displaced by the progeny of Lgr5^+^ cells located at lower rows. By contrast, due to the presence of retrograde movement in SI crypts, cells at the border of the crypt base, possibly including those outside the Lgr5^+^ zone, can take turns to visit the crypt centre, and have therefore a chance to function as long term effective stem cells. Thus, we conclude that stem cell identity is neither cell-intrinsic nor marked by distinct molecular signatures, but rather defined by the potential of cells to be present in or enter a location that both induces self-renewal and prevents displacement.

Our experimental data and mathematical modelling demonstrate the importance of random cell relocation and retrograde movement in governing stem cell identity, and show surprising differences in the rate and dynamics of monoclonal conversion in the SI versus LI, despite strong similarities in crypt characteristics. Our model identifies a single dimensionless parameter, i.e., the ratio between cell repositioning rate and division rate (*k_r_*/*k_d_*), which sets the spatial extent of the effective stem cell region, defined as the length along the axis of the crypt at which cells have a significant probability to contribute to long-term renewal. In the LI, *k_r_*, and thus retrograde movement, is so minimal that self-renewal potential is largely limited to cells positioned at row 0 (i.e., approximately 5-10 stem cells compete laterally and neutrally), whereas more complex dynamics emerge in the SI due to retrograde movements, with neutral competition between cells of the same row^1,2^ as well as biased competition between cells of different rows (stochastic conveyor belt).

Importantly, the differential rate of cell repositioning and retrograde movement was sufficient to explain the differential dynamics underlying regeneration of the stem cell pool, competition for niche space in the SI and LI, as well as the faster clonal evolution and shorter fixation times in the LI compared to the SI. This underlines the importance of a better understanding of the molecular and cellular bases of retrograde movements. Interestingly, we observed less retrograde movement upon Porcupine inhibition while proliferation was unaltered. This was consistent with complementary experiments from *in vitro* and decellularised migration assays, which indicates a role for Wnt signalling in driving active migratory movements even in regions beyond the crypt base with low levels of Wnt.

To induce self-renewal in the Wnt-rich region, the pericryptal stroma is a sufficient Wnt source in the absence of PCs ^18,19^. However, for retrograde movement, this source is insufficient since this movement is not present in the LI. Seemingly, for movements in regions beyond the crypt base, other Wnt sources, such as PC in the SI, are required. Although the low levels of Wnt outside the crypt base may be insufficient to induce self-renewal properties, our data suggest that these levels are high enough to induce migration enabling cells to enter Wnt-rich regions. Since Wnt is known to regulate numerous other processes including induction of self-renewal, it remains difficult to directly provide causality between stem cell function and retrograde movement. Nevertheless, our results suggest that retrograde movements in the SI are not only a result of random repositioning during cell division, but represent a new level of active stem cell regulation that can be experimentally and pharmacologically manipulated. In this regard, it is also interesting that Wnt-antagonists are increased during aging^20^ and early adenoma formation^21^. Given the importance of homeostatic stem cell dynamics in predicting the response of the cellular compartments to non-neutral mutations, retrograde movement discovered here may contribute to susceptibility to pathological conditions such as tumour initiation along the intestinal tract.

## Methods

### Mice and treatment

All animal experiments were carried out in accordance with the guidelines of and approved by the animal welfare committee of the Netherlands Cancer Institute and Hubrecht Institute (KNAW). The following mouse strains were used: Lgr5eGFP-Ires-CreERT2^3^; R26-LSL-tdTomato^22^ and R26-confetti^2^. For lineage tracing experiments, Lgr5eGFP-Ires-CreERT2;R26-Confetti and Lgr5eGFP-ires-CreERT2;LSL-tdTomato mice were used. Lgr5eGFP-Ires-CreERT2 were used to isolate Lgr5^+^ cells and C57/B6 mice were used to decellularise intestines for the decellularised intestine experiment. Lgr5-DTR-GFP^17^ mice were used for the Lgr5^+^ cell ablation experiments. Genentech provided the Lgr5-DTR-GFP mice through their MTA program. Mice (mixed or C57/B6 background) were housed under standard laboratory conditions in SPF cages in an animal room at constant temperature (19-23°C) and regulated humidity under a 12-h light-dark cycle and received standard laboratory chow and water ad libitum. Male and female mice between 8 and 50 weeks of age were used for static lineage tracing and IVM experiments. For whole mount imaging and imaging of isolated crypts, RNA-seq, colony forming assay, decellularized intestine, Lgr5^+^ cell ablation and clone dispersion experiments, intestines from 8–50 week-old male and female mice were used. Strains were bred in house. Lgr5eGFP-Ires-CreERT2;R26-Confetti received 1 mg tamoxifen (Sigma) for IVM lineage tracing experiments and 5 mg tamoxifen (Sigma) for static lineage tracing experiments. For long-term (intravital) tracing, small and large intestine were taken from the same mouse. This was not possible for short-term intravital microscopy experiments, since only either the small or large intestine fitted behind the abdominal imaging window (AIW). For the decellularised intestine experiment, the Porcupine inhibitor LGK974 was administered in a concentration of 5 mg/kg BID (oral) in a vehicle of 0.5% Tween-80/0.5% methylcellulose. Mice of the same litter were randomly assigned to each condition regardless of the sex. Different conditions were imaged and analyzed in a random fashion to minimize potential cofounders.

### Surgery

All surgical procedures were performed under ~2% isoflurane (v/v) inhalation anesthesia. Before and 8-12 hours after surgery, mice were treated with a dose of buprenorphine (subcutaneous, 100 ug/kg mouse, Temgesic; BD Pharmaceutical System). Rimadyl (64 μg/ml, Carprofen; Zoetis B.V.) was given in drinking water for 3 days after the surgery. For short-term intravital microscopy, an AIW was placed as described previously^23^. In short, the left lateral flank of the mouse was shaved and disinfected. An incision was made through the skin and peritoneum of the mouse and a purse string suture was placed along the edge of the wound. The ileum (SI) or cecum (LI) was exposed and a disinfected AIW (>1h in 70% (v/v) ethanol) was placed on top. In the case of the ileum, the mesenterium was fixed to the cover glass using Cyanoacrylate Glue (Pattex) and CyGel (BioStatus Limited) was added on top to prevent liquid accumulation. In the case of the cecum, it was fixed to the titanium ring of the AIW using Cyanoacrylate Glue (Pattex). When these substances were dry, the intestine and AIW were placed back in the abdominal cavity and the skin and abdominal wall were placed into the groove of the AIW. Subsequently, the suture was tightened. After surgery, the mice were closely monitored daily for reactivity, behaviour, appearance and defecation. For repetitive long-term imaging, parts of the intestine that were imaged were exteriorized through a midline abdominal incision. Tissue hydration was maintained by creating a wet chamber, covering the mice with parafilm and the exposed tissue with PBS-drenched gauze. After the imaging session imaged tissue was placed back in the abdomen and the abdomen was closed using vicryl absorbable sutures (GMED Healthcare BVBA).

### Intravital microscopy

For every imaging session, mice were sedated using isoflurane inhalation anesthesia (~1.5% isoflurane/O2 mixture), and placed in a custom-designed imaging box. For short-term imaging, mice were imaged once a day for a maximum of 3 h. For long-term imaging, mice were imaged 2-3 d after label-induction and 8 weeks thereafter. Z-stacks and overview images were recorded using the Navigator function from Leica. The patchy expression pattern of the Lgr5 knock-in allele, in combination with specific landmarks such as blood vessels, allowed repeated identification of imaged areas over consecutive days. After imaging, the acquired images were only if necessary, corrected for bleed through, cropped, smoothened, rotated and contrasted linearly in Fiji (v.2.3.0) (https://imagej.nih.gov/ij/).

### Whole mount preparation

For whole mount imaging, intestines were harvested and the lumen was flushed with ice-cold PBSO. The tissues were opened longitudinally and for the ileum, villi were removed from the luminal surface using a cover glass. The tissues were washed in ice-cold PBSO and fixed for 30 min in 4% formaldehyde solution (w/v) (Klinipath)) or periodate-lysine-4% paraformaldehyde (PLP) overnight at 4°C^24^. For antibody labeling, the tissues were permeabilized in 0.8% Triton X-100 in PBS containing 3% BSA. Subsequently, stretches of ~ 2 cm of fixed tissue, were mounted between 2 coverslips and embedded in Vectashield HardSet Antifade Mounting Medium (Vector Laboratories). Crypts were imaged from the bottom using the same equipment and settings as for intravital microscopy described below. For storage, PLP-fixed tissues were incubated in sucrose for >6 hours and frozen in OCT at -80°C.

### Crypt isolation

For crypt isolation, intestines were harvested and the lumen was flushed with ice-cold PBS. Tissue was opened longitudinally, villi were removed from the luminal surface of distal ileum. Parts of approximately 3 cm of ileum and intact but opened cecum were incubated with 30mM in EDTA in HBSS at room temperature for 20 minutes. After vigorously shaking, the release of the epithelium from the mesenchyme was checked using a microscope. Suspensions were filtered (100um) before spinning down (5 minutes at 4°C, 88 rcf). Pellets containing isolated crypts were washed with cold PBS, fixed in 4% PFA (30 minutes at room temperature), permeabilised in 1% triton X-100 (45 minutes at room temperature), blocked in blocking buffer for 30 minutes at room temperature (1% BSA, 3% horse serum, 0.2% Triton X-100 in PBS) before antibody labeling.

### Cell proliferation and antibody labeling

To label cells in S-phase, 1 mg of 5-ethynyl-2-deoxyuridine (EdU, 200 ul in PBS) or 2 mg bromodeoxyuridine (BrdU, 200 ul in PBS) was injected intraperitoneally 2 or 4 hours prior to sacrifice (indicated in figure legends). Tissues were processed for whole mount analysis or crypt isolation as described above. Click-it staining reaction was performed according to the manufacturer’s protocol (Click-it EdU, ThermoFisher/invitrogen). For labeling of BrdU incorporation, crypts were incubated in 2N HCl at 37°C for 15 minutes to denature the DNA followed by 15 minutes in 0.1 M sodium borate for neutralization before incubation with BrdU antibody (1:50 Abcam, 6326) and GFP-antibody (1:200 Abcam, 6673) overnight. To label cells in mitosis phospho-Histone H3 antibody was used (1:200, Millipore, 06-570). To visualise Ephrin B2 and B3, intestines were stained with Ephrin B2-antibody (1:100 R&D system, AF467) and Ephrin B3-antibody (1:100 R&D system, AF432). Stainings were finalized by incubation with Alexa Fluor secondary antibodies, donkey anti-goat IgG Alexa Fluor 488 (Cat# A-11055), donkey anti-rabbit IgG (H+L) Alexa Fluor 568 (Cat# A-10042) and chicken anti-rabbit IgG (H+L) Alexa Fluor 647 (Cat#A-21443) (1:200 Invitrogen) combined with DAPI followed by mounting in antifading mounting medium (Vectashield, Vector laboratories). To visualise Wnt target gene expression, formalin-fixed intestinal tissue was incubated with the following Primary antibodies as previously described^21^: CD44 (1:50 BD Biosciences #550538), Cyclin D1 (1:50 Dako #M3635).

### RNA in situ hybridisation

In situ hybridisation for *Lgr5* (#312178), *Smoc2* (#318548), *Ascl2* (#412218) and *Axin2* (#400338) mRNA (all from Advanced Cell Diagnostics) was performed using RNAscope 2.5 LS Reagent Kit–BROWN (Advanced Cell Diagnostics) on a BOND RX autostainer (Leica) according to the manufacturer’s instructions.

### Microscopy equipment and settings

Tissues were imaged with an inverted Leica TCS SP8 confocal microscope. All images were collected in 12 bit with 25X water immersion objective (HC FLUOTAR L N.A. 0.95 W VISIR 0.17 FWD 2.4 mm).

### Sample preparation for RNA-seq and colony formation assay

Small and large intestinal crypts were isolated as previously described^25^. To obtain single cells, crypts were treated with TrypLE (Life Technology) supplemented with 30 μg/mL DNaseI (Sigma) and Y-27632 (10 μM) (incubation at 37 °C for 30 minutes). Dissociated cells were filtrated by 100-μm cell strainer (Greiner Bio-One), and cells were resuspended and incubated with antibodies for 30 minutes on ice in 1 mL PBS containing FCS 5%, EDTA 5mM (Accugene, Lonza), B27 2% (Thermo Fisher Scientific, cat. no. 17504-044), N-acetylcysteine 1.25 mM (Sigma-Aldrich, cat. no. A9165), mEGF 50 ng/ml (Peprotech, cat. no. 315-09), Noggin and R-spondin1 both 10% (conditioned medium prepared in house), Y-27632 (1 μM) and 4 μg/mL DNaseI (Sigma). The cell suspension was filtered by 70-μm cell strainer (Celltrix) and subjected to flow cytometry on FACS Aria Fusion (BD Biosciences). Dead cells were eliminated by gating of forward/side scatter, forward/pulse-width parameters, and negative staining for 7-AAD (eBioScience). Lgr5^+^ cells were sorted by positive staining for Epcam (CD326) (BD Bioscience, Cat #563214) and endogenous Lgr5-GFP signal. Data were visualised using FlowJo 10.6.1 (https://www.flowjo.com/).

For RNA sequencing, sorted cells were collected into 1.5-mL tubes containing FACS buffer, washed with PBS, pelleted and stored at -80°C for further processing. RNA was isolated using RNeasy Micro Kit (Qiagen).

For colony formation assay, sorted cells were collected in FACS buffer, washed with PBS, pelleted and embedded in Cultrex PathClear Reduced Growth Factor Basement Membrane Extract Type 2 (BME2) (Amsbio, 3533-005-02), followed by seeding on 24-well plate (500 cells in 20 μL of BME2 per well). The number of organoids were counted 7 days after seeding.

### Processing RNA-seq expression data

For library preparation, the SMART-Seq Stranded Kit (Takara) was used. Sequencing was performed by CeGaT on NovaSeq 6000 with 100bp paired end reads. Demultiplexing of sequencing reads for all 18 samples (3 biological replicates of Lgr5^+^ high, medium and low cells for both SI and LI) was performed with Illumina bcl2fastq (version 2.20) where adapters were trimmed with Skewer (version 0.2.2)^26^. Trimmed raw reads (average length 96-102 nucleotides) were aligned to genome mm10 using STAR (version 2.5.2b)^27^ for genome assembly and gene count.

Differential expression analysis was performed between groups considering biological replicates of intestinal locations using DeSeq2 (version 1.34 in Bioconductor 3.14)^28^ in R (version 4.1.1) (R Core Team 2021). After normalization, all regions with mean count greater than zero were included to improve detection power. Mean count across genes above zero was selected based on the standard deviation for each genes. With normalised counts we calculated the log2 fold change and obtained the p-value (Wald test) and p adjusted with Benjamin-Hochberg correction for multiple testing.

Relationship between intestinal locations based on gene expression were visualised through principal component analysis (PCA). We used regularized logarithmic transformation (rlog) of the normalised counts for all samples to obtain the PCA so each gene contributes equally to the distance between samples. Volcano plots were obtained in R only highlighting differentially expressed genes with a p value < 0.001 and a log2 fold change higher or lower than 2 and -2 for up-regulated and down-regulated respectively.

### Preparation of decellularised mouse intestine

Small intestinal tissue was decellularised as previously described by^15^. Pieces of decellularised small intestinal ECM (dECM) were washed 10 min in PBS, plated on a culture dish, and stained with Col-F Collagen Binding Reagent dye (Col-F) (BioSite, 260-6346) overnight at 4°C. dECM was then washed with fresh PBS, and primed for approximately 30 minutes with live imaging medium consisting of Advanced DMEM/F12, 1x penicillin/streptomycin, 1x Glutamax (Thermo Fisher Scientific), 10mM HEPES (Thermo Fisher Scientific), 1x B27 (Life Technologies), 1x N2 (Life Technologies), 1mM N-acetylcysteine (Sigma), 50ng/ml of murine recombinant Epidermal growth factor (R&D), 100ng/ml recombinant murine Noggin (Peprotech), 10μM Y-27632 (Sigma) and 1 μM Jagged-1 peptide (Anaspec). After priming, excess medium was discarded to allow seeding of Lgr5^+^ cells.

### Single cell isolation and FACS

Lgr5^+^ cells were isolated as previously^20^, with a modified protocol to effectively detach epithelium. Briefly, longitudinally opened intestine was cut into 5-7 cm pieces, and incubated in 10mM PBS-EDTA for 30 min. Intestinal pieces were first gently scraped using a microscopic slide to discard villous material and scraped once more to collect crypts.

### Live imaging of Lgr5^+^ cells on dECM

Lgr5^+^ cells were stained and washed with 5uM 647CellTracker (Invitrogen) according to manufacturer’s protocol. Approximately 10 000 cells in 10-20μl of medium were then seeded on to the scaffold and were allowed to settle for 10 minutes before adding 10μl of medium. Additional medium was then carefully introduced in two phases: 20μl at 20 minutes after seeding, and 260μl at 30 minutes after seeding.

Cells were live imaged with a Nikon spinning disk confocal, using a PlanApo 10×/0.5 NA dry objective and the NIS-Elements AR 5.02.01 software (Nikon). A stack of images covering the whole height of the crypt-villus axis was captured with 2,5μm separation between images, and full stack was imaged every 2 minutes. Cells were imaged for about 4 hours and live/dead cells were assessed at the end of imaging by staining with DAPI (Thermofisher, 1μg/ml). Col-F was detected by using a 488 nm laser with a 520/30 emission filter, the 647CellTracker was detected using a 640 nm laser with a 685/40 emission filter, and a 405 laser with an emission filter 447/60 for DAPI.

### Analysis of single Lgr5^+^ cell migration on dECM

For preprocessing the images were corrected for x-y drift, when needed, with “Register Virtual Stack Slices” and “Transform Virtual Stack Slices” plugins in ImageJ. In short, the brightfield images were used to make a maximum intensity projection over the z-levels. Next, drift was registered using with the following parameters: “Feature extraction model: Translation”; “Registration model: Translation --no deformation”; “Save transforms” option selected. Subsequently, the transformations over the time lapse were applied for each of the respective z-level on the brightfield and fluorescent images using the “Transform Virtual Stack Slices” plugin, which were then reassembled into hyperstacks.

Cell tracking was performed with a combination of automatic tracking, using the “Trackmate” plugin (v6.0.3)^29^ in ImageJ, and manual adjustment of the selected tracks (e.g., joining split tracks as needed). The results were then exported for subsequent analysis.

Analysis of the cell tracking results was performed using custom scripts in Python (v3.10). In short, each cell was classified based on its location in z (acquired by visual inspection of the decellularised intestinal pieces before the start of the time lapse) into crypt or villus regions. Cell motility was calculated as Euclidean distance between each timepoint and its preceding timepoint. Finally, results were plot as the mean and 95% CI, using a polynomial fit (numpy.polyfit, numpy version 1.19.5) for each of the tracks.

### *In vitro* migration assay

Small intestinal organoids from the Lgr5-EGFP-ires-creERT2 × R26R-confetti mouse were generated and directly induced (i.e., before the first passage) with TAT-Cre recombinase (5uL/mL, MERCK #SCR508). After 5 days in culture both the Lgr5^+^/confetti-RFP^+^ and Lgr5^+^/confetti-YFP^+^ stem cells were sorted (SONY MA900 cell sorter) and plated as single cells^30^. After 7 days of culture, 2500 or 1250 Lgr5-GFP^+^;confetti-RFP^+^ stem cells and 1,250 CD24^hi^/confetti-YFP^+^ Paneth cells were sorted from these two respective organoid cultures^14^ into Ultra-low attachment 96-well round-bottomed plates (Sarstedt, #82.1582.001) containing 135uL of medium. Paneth cells were sorted based on CD24 expression (1:400, Thermo Scientific #48-0242-82) and all cells were exposed to live-dead dye (1:200, DRAQ7, Thermo Fischer Scientific #D15105) to sort for living cells. The basic medium^25^ is supplemented with either 50% Wnt3A CM, IWP2 (2μM, Tocris 3533)^31^. After sorting the plate was left on ice for 15 min and 15uL of Matrigel was added before 5 min of centrifugation (300*g* with low acceleration and breaking). The fluorescent and brightfield images were acquired every 15 min by a spinning disk confocal (ImageXpress Micro Confocal) equipped with a 10x (NA = 0.45) Nikon Plan Apo Lambda objective and live imaging chamber (humidified with sterile water and maintained at 37 °C, 6.4% CO_2_). All organoid lines were tested and confirmed negative for mycoplasma contamination.

### Processing of *in vitro* migration data

TrackMate plugin (Imagej version 2.3.0)^29,32^ was used to unbiasedly track the location of individual cells over time (estimated object diameter = 10 μm, linking max distance = 45 um, gap closing max distance = 15 um, gap closing max frame gap = 1). CelltrackR^33^ (R version 4.1.1), DiPer^34^ (Microsoft Excel 2016), and custom made code were used to calculate mean square displacement, speed, and persistence. Replicates with low number of tracks (<40) or where the cell density was near confluent were excluded from the analysis, leading to exclusion of one biological replicate. For each condition, the data is based on 3 independent biological replicates, each including 2 to 4 technical replicates. To calculate the migration speed and directionality of the motile cells, only tracks from motile cells were included (i.e. with speed >0.3 μm/min).

### Diphteria Toxin treatment

For the Lgr5^+^ cell ablation studies, male and female Lgr5DTR:eGFP mice received 50 μg kg^-1^ Diphtheria Toxin (DT) in PBS through intraperitoneal injections for two days in a row and were sacrificed after 24 hours, 48 hours, 96 hours, 7 days and 15 days. Small and large intestines were collected, stained with DAPI and Phalloidin Alexa Fluor 647 (ThermoFischer Scientific, cat# A22287) and imaged *ex-vivo*.

### Statistics

Any a priori sample size calculation could not be performed, since the effect size and the variance was not known before the experiments. No data or animals were excluded, except for the *in vitro* migration assay, where one biological replicate was excluded due to low number of trackable cells (<40). Due to differences in crypt morphology, blinding was not possible when comparing small and large intestine. All other comparisons of intravital microscopy experiments and the *in vitro* migration assay were analyzed in a blinded fashion. The imaging data were randomized by one researcher and analyzed by another researcher. For the analysis of the migration data, the randomization was done by custom script in Python and R, and analyzed blinded by the researcher. All P-values were calculated using a two-sided Mann-Whitney U test in GraphPad Prism v9 (GraphPad Software, LA Jolla, CA). See Supplementary Theory for details on statistics concerning the mathematical modelling.

## Extended Data

## Supplementary Material

Movie 1

Movie 2

Supplementary Figure 1

Supplementary Note

## Data Availability

The RNA sequencing data is available through the Gene Expression Omnibus (GEO) with the accession code GSE194250. All other data are included within the paper, Extended Data and Supplementary Information and are available through the online data sharing platform Figshare: https://figshare.com/projects/Azkanaz_et_al_2022_Retrograde_movements_determine_effective_stem_cell_numbers_in_the_intestine/139210. Source Data for all figures are provided with this paper.

## Code availability

The codes and data used for fits, simulations, RNAseq, *in vitro* cell migration and single Lgr5^+^ cell migration on dECM are available in GitHub (https://github.com/JaccovanRheenenLab/Retrograde_movement_Azkanaz_Nature_2022).
