# Supplementary figures and images for: Retrograde movements determine effective stem cell numbers in the intestine
Source: Nature. Author manuscript; Available in PMC 2023 Aug 8. (PMC7614894; doi:10.1038/s41586-022-04962-0)

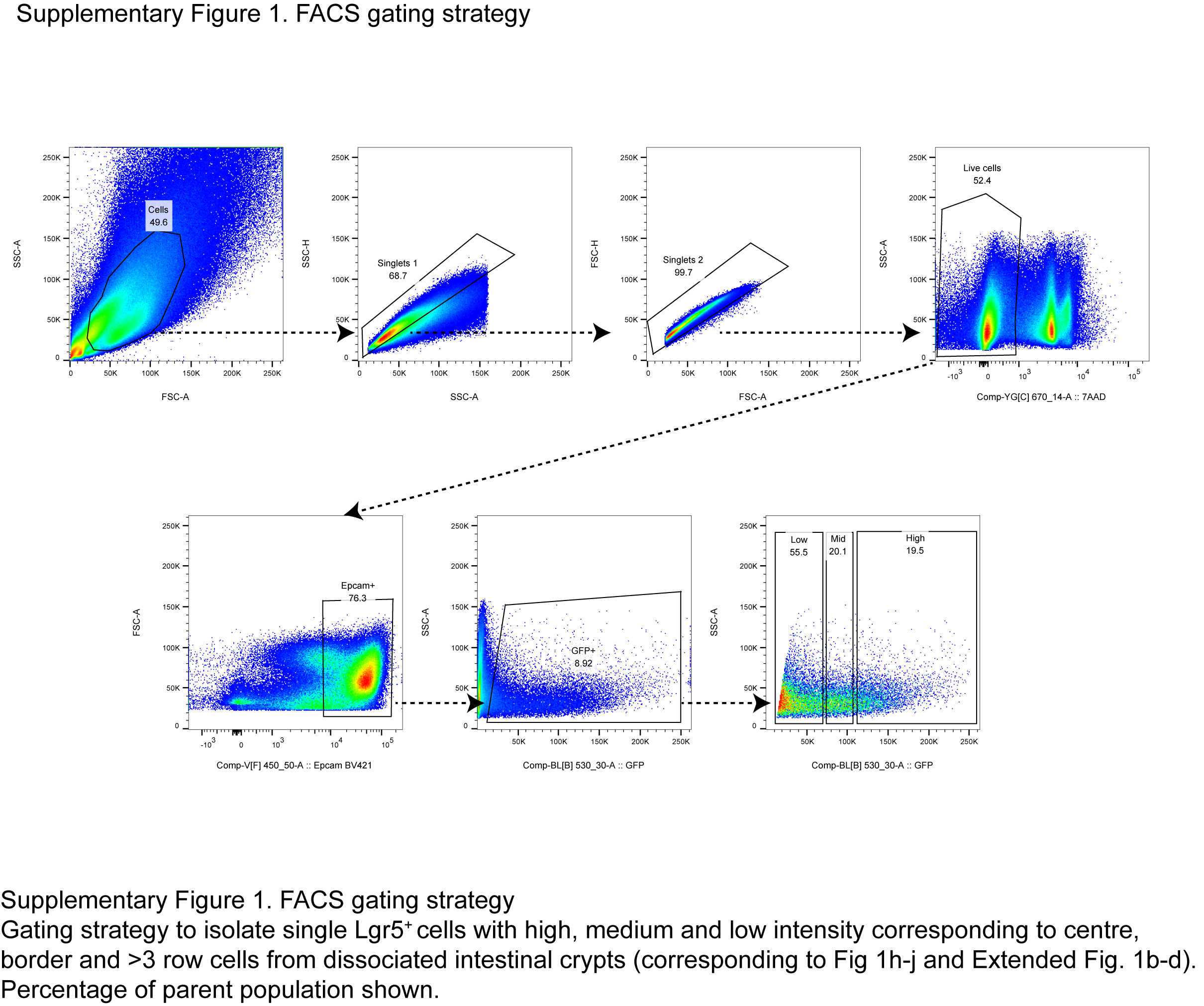

Supplement: Supplementary Figure 1 [file EMS182587-supplement-Supplementary_Figure_1.tiff]
