# Supplementary material for: Retrograde movements determine effective stem cell numbers in the intestine
Source: Nature. Author manuscript; Available in PMC 2023 Aug 8. (PMC7614894; doi:10.1038/s41586-022-04962-0)
Supplement: Supplementary Note [file EMS182587-supplement-Supplementary_Note.pdf]

SUPPLEMENTARY NOTE

Contents

1. Basics of stochastic conveyor belt (SCB) dynamics 1
1.1. Biological motivation for the modelling 1
1.2. Formal definition of the SCB dynamics 2
1.3. SCB dynamics in real crypts 8
1.4. Nature of the stochastic rearrangements and retrograde movements 10
2. Numerical simulations of the 2D SCB dynamics and statistics 11
2.1. Details on the implementation of 2D simulations 11
2.2. Statistical approach for fitting and confidence intervals 11
2.3. Clonal fragmentation from fixed data 14
2.4. Timescale of monoclonal conversion 15
2.5. Dynamics of filling after ablation 15
3. Comparison to previous models 16
Supplementary references 17

In this supplementary note we provide additional details on the modelling approach used in the manuscript, as well as details on the numerical and statistical tools used to analyze the data.

1. Basics of stochastic conveyor belt (SCB) dynamics

1.1. Biological motivation for the modelling.

The intestinal epithelium is renewed from proliferative intestinal stem cells at the bottom of crypts (Fig. 1a), while cell loss occurs at the tip of villi. The push up force exerted by the duplication of stem cells creates a constant, unidirectional flow of cells from lower to upper levels along the crypt-villus axis. This continuous advection generates a conveyor belt-like dynamics within the epithelial sheet. If one only considers advection and we set ourselves in a one-dimensional setting, the question of whether a cell initially located at a given position can give rise to the lineage that eventually colonizes the whole system has a simple answer: the lineage from the cell at the bottom-most row of the crypt will colonize the whole system with probability 1. Indeed, any cell located in another level has zero probability to survive long-term, as the push up force triggered by cell divisions creates a unidirectional drift towards the villus. For that reason, one may refer to such a dynamics as a deterministic conveyor belt. If we consider a more realistic geometry, the bottom level is populated by several cells that compete laterally and neutrally for lineage survival [1, 2]. Such dynamics

at the bottom of the crypt (in the lateral/circumferential direction) is well-described by a 1D Voter model on a ring with an estimated amount of cells around $12-16$. Subsequent data and modelling from continuous labelling [3] proposed a smaller number of functional stem cells, around $5-7$ stem cells per crypt.

But how is this number of stem cell determined in the first place from a biological perspective? One definition would be molecular, with Lgr5+ being a marker of stemness that is expressed within the first four bottom-most cell rows. However, it has been reported via short-term live-imaging that in the SI, bottom vs top layers of Lgr5+ cells have highly different survival dynamics, which was explained via short-term advantages linked to location [4]. However, whether and how this dynamics is conserved in different intestinal regions remains unclear, together with what determines the strength of the competition for space. Here, we combine short-term and long-term live-imaging, to help bridge the time scale of individual cell movements with the time scale of monoclonal conversion (and stem cell competition). This provides insights into how the effective number of stem cells per crypt is defined, understanding the effective number of stem cells as the amount of cells giving rise to long term clones. Interestingly, this framework is able to identify surprising differences between Large and Small intestines (resp. denoted subsequently as LI and SI) .

In particular, the modelling approach is based on two key experimental findings: i) a stochastic addition to the classical "conveyor belt" picture, allowing cells to randomly reposition to more favourable locations, ii) the strength of stochasticity/repositioning varies drastically between the SI and the LI despite strong anatomical similarities. This empirical observation of retrograde movements implies that other factors, besides the one-directional drift induced by cell duplication, must be taken into account. The constraint of homeostasis/constant cellular density in time puts strong constraints on retrograde movements. Indeed, any downward active migration of a given cell i must be compensated by the upward migration of a given cell j by the same amount. Importantly however, such events of active cell migration or cells stochastically switching their positions can counter-act the drift induced by cell divisions, allowing cells from upper levels to relocate in more favorable positions. In Fig. 2e of the main text we schematize this dynamics. In that case, the question whether a cell located at a given level can colonize the whole organ becomes a complex problem, and we provide in this Supplementary Note details on the modelling and statistical approach used to interpret the data [5]. Finally, although we find evidence (see main text for details) for relocations to rely on Wnt-dependent active cell migration, the presented theoretical framework is quite insensitive to the particular details through which they occur (as detailed below).

1.2. **Formal definition of the SCB dynamics.**

1.2.1. *1D SCB dynamics.*

We will first define the dynamics in a 1D setting along the crypt-villus flow direction, to get insights into how the row of a given cell is located influences the survival probability of its lineage. We consider a column of cells arranged in a finite segment $[0, L]$, in which the length unit is scaled to the average diameter of a cell. We assume a mean field approach in which each cell divides at constant rate k_d. When a new cell is born at longitudinal level j, it moves either one position left or right, pushing the old neighbour cell to level $j+1$, or directly up, pushing the cell that was immediately above the mother cell from level $j+1$ to level $j+2$

(See Fig. 2e for a schematic view). This mechanism ensures that the density of cells remains constant, as required by the constraint of homeostasis. This division process creates a positive push up force projected through the longitudinal axis of the organ. When a cell reaches level L and is pushed, it disappears from the system. In addition, the position of the cells can fluctuate stochastically at rate k_r in either direction. The combination of the advection force due to cell duplication at rate k_d and stochastic rearrangements occurring at rate k_r create a dynamics that can be abstracted as a conveyor belt with random relocations, defining the essentials of the *Stochastic Conveyor Belt* (SCB) dynamics. Note that, in the case where $k_r = 0$, we recover the standard scenario of a deterministic conveyor belt as outlined in the previous section.

To study lineage dynamics, we first need to define the SCB dynamics at the single cell level. According to the description above, the position of cell in cell-unit lengths from the bottom of the organ will be a random variable X subject to an advection force due to duplication $\sim k_d X$, since X cells will duplicate *below* at every time unit, and a random fluctuation of amplitude $\sqrt{k_r}$. In the continuous approximation, the stochastic differential equation for the positional variable X will be thus [5]:

$$dX = k_d X dt + \sqrt{k_r} dW \quad , \tag{1.1}$$

where dW is the Wiener or Brownian differential [6, 7, 8]. According to the above dynamics, the probability that a cell that started at position n is at a given position x of the organ at time t is governed by the following Fokker-Planck equation [7, 8]:

$$\frac{1}{k_d}\frac{\partial p_n}{\partial t} = -\frac{\partial}{\partial x}(x p_n) + \frac{k_r}{2k_d}\frac{\partial^2 p_n}{\partial x^2} \quad .$$

Accounting for the division rate implies the addition of a reaction term, that is, the cell, aside the random fluctuations provided above, also divides at a certain rate. The cell that at $t = 0$ was at position n will define a lineage, c_n, made up of all its descendants. In the continuous setting, such a lineage will be described as a density ρ_n of cells of the lineage c_n that will depend both on the position and the time. This will lead to a reaction-diffusion equation governing the density of lineage c_n under the SCB dynamics that reads:

$$\frac{1}{k_d}\frac{\partial \rho_n}{\partial t} = -\frac{\partial}{\partial x}(x \rho_n) + \frac{k_r}{k_d}\frac{\partial^2 \rho_n}{\partial x^2} + \rho_n \quad . \tag{1.2}$$

While this equation has no steady state solution, its time-dependent solution can be derived. We follow the steps provided in [5].

1.2.2. *Interpretation of* $\rho_n(x, \tau)$.

Eq. (1.10) must be interpreted carefully: it is not telling us what is the *physical* density of cells of a given lineage, but the *relative strength* of such a lineage at a given point and time in the competition with the other cells to occupy a given position at a given time. That is: in an ensemble picture, the probability of finding a cell of lineage n at position x at time t will depend on the relative strength of $\rho_n(x, \tau)$ with respect to other densities. Essentially, we are circumventing the fact that there is only one cell per position, and we are projecting such constraint at the ensemble level as the result of a competition where the possibilities to win are proportional to $\rho_n(x, \tau)$.

1.2.3. *Solutions of the 1D SCB equation.*

To solve Eq. (1.2) we impose as initial condition that $\rho_n(x,0) \sim \mathbf{N}(n,1/2)$, i.e., a normal distribution centred at n with variance 1/2. This condition describes a single cell located at position n as a density that spreads significantly only at $n \pm 1/2$ and whose integral is equal to 1, as is expected for a single clonally labelled cell. Natural boundary conditions apply [8]. The solution of Eq. (1.2) factorizes as follows:

$$\rho_n(x,t) \propto \phi_1(x,t)\phi_2(t) \quad , \tag{1.3}$$

where $\propto$ implies "up to some multiplicative positive constant" and ϕ_1 is the solution of:

$$\frac{\partial \phi_1}{\partial \tau} = -\frac{\partial}{\partial x}(x\phi_1) + \frac{k_r}{2k_d}\frac{\partial^2 \phi_1}{\partial x^2} \quad , \tag{1.4}$$

and ϕ_2 is the solution of:

$$\frac{\partial \phi_2}{\partial \tau} = \phi_2 \quad .$$

which has a simple solution:

$$\phi_2 \propto e^{\tau} \quad , \tag{1.5}$$

where we have defined for convenience the rescaled time unit $\tau = k_d t$. Thus, ϕ_2 represents the fact that cells proliferate at rate k_d so that the entire lineage increases in size exponentially regardless of its position.

Computing ϕ_1, we first observe that (1.4) is a Fokker-Planck like equation for the time evolution of the probabilities of a random variable following a mixture of Brownian motion with a given amplitude k_r/k_d and a drift parameter $x\phi_1(x,\tau)$. Equation (1.4) has its stochastic differential equation counterpart in:

$$dX = Xd\tau + \sqrt{\frac{k_r}{k_d}}dW \quad , \tag{1.6}$$

which is equivalent to Eq. (1.1). Recall that Eq. (1.6) explicitly tracks the real trajectory of a cell (and not lineage prevalence. The above described stochastic process has no stationary solutions, which is in agreement with the SCB dynamics: all cells will sooner or later be pushed out from the system. Imposing the following initial conditions $t_0 = 0$, $\phi_1(x,0) \sim \mathbf{N}(n,1/2)$ and natural boundary conditions, we observe that:

$$d\left(e^{-\tau}X(\tau)\right) = dX(\tau)e^{-\tau} - e^{-\tau}X(\tau)d\tau \quad .$$

Then, multiplying both sides of Eq. (1.6) by $e^{-\tau}$, and after some algebra, one finds that:

$$d\left(e^{-\tau}X(\tau)\right) = \sqrt{\frac{k_r}{k_d}}e^{-\tau}dW \quad ,$$

leading to:

$$X(\tau) = x_0 e^{\tau} + \int_0^{\tau}\sqrt{\frac{k_r}{k_d}}e^{(\tau-s)}dW \quad . \tag{1.7}$$

The integral is a standard stochastic integral with respect to a Wiener process. According to *Ito's isometry* [6] one has that the law governing the random variable described by the integral is a normal distribution

$\mathbf{N}(0, \sigma^2(\tau))$. In our case this reads:

$$\int_0^\tau e^{(\tau-s)}\sqrt{\frac{k_r}{k_d}}dW \sim \mathbf{N}\left(0, \int_0^t \left|\sqrt{\frac{k_r}{k_d}}e^{(t-s)}\right|^2 ds\right) \quad ,$$

which means that the explicit form of $\sigma^2(\tau)$ is thus given by:

$$\sigma^2(\tau) = \int_0^\tau \left|\sqrt{\frac{k_r}{k_d}}e^{(\tau-s)}\right|^2 ds = \frac{k_r}{2k_d}\left(e^{2\tau}-1\right) \quad . \tag{1.8}$$

Finally, from Eq. (1.7), we conclude that the time dependent mean, $\mu(\tau)$, is:

$$\mu(t, x_0) = x_0 e^\tau \quad .$$

That leads to:

$$\phi_1(x,\tau) \propto \sqrt{\frac{k_d}{2\pi k_r\left(e^{2\tau}-1\right)}}\exp\left\{-\frac{k_d}{2k_r}\frac{(x-ne^\tau)^2}{(e^{2\tau}-1)}\right\} \quad . \tag{1.9}$$

According to Eqs. (1.3, 1.5) and (1.9), the solution of the SCB equation (1.2) can be fairly approximated as:

$$\rho_n(x,\tau) \propto \sqrt{\frac{k_d}{2\pi k_r}}\exp\left\{-\frac{k_d}{2k_r}\left(\frac{x-ne^\tau}{e^\tau}\right)^2\right\} \quad . \tag{1.10}$$

1.2.4. *Clone survival probability.*

According to the definition of $\rho_n(x,\tau)$, the probability for a lineage n to survive after time τ, $p(c_n,\tau)$ will be defined in relation to $p(c_0,\tau)$ as:

$$\frac{p(c_n,\tau)}{p(c_0,\tau)} = \int_0^L \rho_n(x,\tau)dx\left(\int_0^L \rho_0(x,\tau)dx\right)^{-1} \quad . \tag{1.11}$$

$p(c_0,\tau)$ is taken as a reference since:

$$(\forall n, \tau > 0)\; p(c_0,\tau) \geq p(c_n,\tau) \quad .$$

The absolute values of these probabilities can be computed numerically by simulating the SCB dynamics [see section 3 of this Supp. Note for details]. We observe that, $\rho_n(x,\tau)$ tends to a constant $\rho_n(\infty) > 0$, defined as:

$$\rho_n(\infty) = \lim_{\tau\to\infty}\rho(x,\tau) \quad ,$$

which is independent of x, taking the explicit form of:

$$\rho_n(\infty) \propto \exp\left\{-\frac{k_d}{2k_r}n^2\right\} \quad .$$

In consequence, one has that:

$$\lim_{\tau\to\infty}\int_0^L \rho_n(x,\tau)dx = L\rho_n(\infty) \quad .$$

Therefore, the probability that lineage c_n occupies the whole organ satisfies the following ratio:

$$\frac{p(c_n,\infty)}{p(c_0,\infty)} = \exp\left\{-\frac{k_d}{2k_r}n^2\right\} \quad . \tag{1.12}$$

Since we know that the whole system becomes monoclonal at large times, we can impose the normalization:

$$\sum_{n\leq L} p(c_n,\infty) = 1 \quad .$$

Leading us to a discrete Gaussian distribution:

$$p(c_n,\infty) = \frac{1}{Z}\exp\left\{-\frac{k_d}{2k_r}n^2\right\} \quad , \tag{1.13}$$

consistent to what is found in [5], and where the normalization constant Z is defined as:

$$Z = \frac{1}{p(c_0,\infty)} = \sum_{n\leq L}\exp\left\{-\frac{k_d}{2k_r}n^2\right\} \ , \quad p(c_0,\infty) = \rho_0(\infty) \quad .$$

According to Eq. (1.10):

$$\rho_0(\infty) = \sqrt{\frac{k_d}{2\pi k_r}} \quad ,$$

as expected for a Gaussian distribution centred at 0 and with $\sigma^2 = k_r/k_d$.

1.2.5. *Generalization of the SCB dynamics to higher dimensions and different geometries.*

To properly study the SCB dynamics in more general geometries, let us consider a Riemannian manifold equipped with a metric tensor $\mathbf{g}$, with components g_{ij} [9]. The property of *local flatness* is assumed [9, 10]. Roughly speaking, this implies that, for small enough regions of the manifold, the geometry has euclidean properties. Let us consider that the push up force due to proliferation has an origin (equivalent to the center of the crypt in intestine) and is projected along the direction of a single coordinate z (the crypt-villus axis in intestine). The general expression of the drift term will be described by the function $v(z)$, defined as the local speed of a cell at position z due to the proliferation of cells located at $z' < z$:

$$v(z) \equiv \frac{dz}{dt} \quad . \tag{1.14}$$

In the case of a 1-dimensional system, as the one described by Eq. (1.2), one has that $v(x) = k_d x$. The surface/volume units are given such that an average cell has a surface/volume of 1 in the corresponding units. Consider the starting position of our cell to be z_n along the coordinate z. If the other coordinates of the manifold are $x_1,...,x_{n-1}$, the surface/volume encapsulated *below* position z_n is given by:

$$S_n = \int ... \int_0^{z_n} \sqrt{g}dx_1...dx_{n-1}dz \quad , \tag{1.15}$$

where g is the determiner of the metric tensor, i.e.:

$$g = \begin{vmatrix} g_{11} & g_{12} & . & . & . \\ g_{21} & g_{22} & & & \\ . & & . & & \\ . & & & . & \\ . & & & & . \end{vmatrix}$$

Cells are assumed to divide at rate k_d. That implies that $k_d S_n$ new cells will be produced *below* the cell located at z_n. This will create an extra surface/volume per time unit of:

$$\frac{dS_n}{dt} = k_d S_n \quad ,$$

that will project into the coordinate z. Using that:

$$\frac{dS_n}{dt} = \frac{dS_n}{dz}\frac{dz}{dt} \quad ,$$

and, then, Eq. (1.14), one can find the general expression for this projection, which reads:

$$v(z_n) = k_d \left(\frac{dS_n}{dz}\right)^{-1} S_n \quad . \tag{1.16}$$

Local flatness enables us to consider that, locally, random fluctuations due to k_r may occur in all directions over a flat geometry, either isotropically or not. For the study of lineage prevalence as a function of the position z only the projection of such fluctuations over the coordinate z matters, since the other contributions cancel out. We call such a projection of the stochastic fluctuations $k_r(z)$.

According to the above results, the general equation for the evolution of cell lineage prevalences along the coordinate z will read:

$$\frac{\partial \rho_n}{\partial t} = -\frac{\partial}{\partial z}(v(z)\rho_n) + \frac{k_r(z)}{2}\frac{\partial^2 \rho_n}{\partial z^2} + \rho_n \quad . \tag{1.17}$$

1.2.6. *Effective number of stem cells emerging from the SCB dynamics.*

In many cases of relevance, probabilities of lineage prevalence under SCB dynamics display a well defined scale for the fluctuations leading to effective retrograde movements. The strength of these fluctuations will define the stem cell number as *the amount of cells being at lower distance to the origin than the typical length of fluctuations over time scale at which the competition resolves.* However, one has to bear in mind that the approach presented here does not assume any intrinsic changes in cell properties, so the *effective* stem cell number will be linked to the probability cut-off one wishes to use (i.e. the minimal probability of lineage survival to consider a cell to be an effective stem cell).

In the simplest 1D case, since $\sigma \sim \sqrt{\frac{k_r}{k_d}}$, and given that $k_r = 0$ implies the existence of 1 stem cell, one can conclude that the effective stem cell number, N_s, can be defined as:

$$N_s \approx 1 + a\sqrt{\frac{k_r}{k_d}} \ , \quad a = 1, 2, 3... \quad , \tag{1.18}$$

where the parameter a depends on the cut-off probability used to define functional stem cells (defining the number of variances away from the mean: if $a = 1$, N_s would be the amount of cells whose lineages persist in the 68% of the monoclonal conversions, if $a = 2$, N_s will refer to the amount of cells whose lineages participate to the 95.5% and so on).

Now we consider a more general case. Consider that, as we did above, that tissues can be abstracted as a n-dimensional $(x_1, ..., x_{n-1}, z)$ Riemannian manifold equipped with a metric tensor $\mathbf{g}$ where the local flatness property holds. Assume there is an origin from which the push up force due to proliferation is exerted along the positive direction of a single coordinate z, and that the lineage survival probabilities associated to the SCB equation (1.17) for this particular case have a bounded σ. An effective stem cell is defined as any cell within a distance $a\sigma$, $a = 1, 2, ...$, of the niche center, meaning that, there is a *belt* containing

$$\sim a\sigma + 1 \quad ,$$

cells from the origin of coordinate z to the last cell that is inside the stem-cell region, considering that $a = 1, 2, ...$ determines the confidence interval. Assuming that we have a well defined and bounded σ, the effective number of stem cells emerging from the SCB dynamics in general geometries, assuming a certain confidence interval $a\sigma$, $N_s(a)$, will be:

$$N_s(a) \approx \int ... \int_0^{a\sigma+1} \sqrt{g} dx_1 ... dx_{n-1} dz \quad . \tag{1.19}$$

1.3. **SCB dynamics in real crypts.**

1.3.1. *Crypt geometry and experimental data structure.*

In the real crypt geometry, the cells are not organized along a one-dimensional line, but are arranged in a 2D surface, that can be approximated by an hemisphere connected to a cylinder (see Fig. 2e and Extended Data Fig. 9a–d). In this section, we show how to adapt the SCB dynamics to this specific geometry. The experimental data was acquired, following the literature, by assessing the clonal dynamics of cells as a function of their cell row/height (resp +0, +1, +2, +3). Importantly, in a hemispherical geometry, the number of cells per row is expected to be approximately constant (see Extended Data Fig. 9d), as seen in the data. Indeed, considering sections of hemispheres at different heights, $\ell_0, \ell_1, \ell_2, \ell_3$, such that the difference between successive heights is constant, $\Delta\ell$ gives rise to identical surfaces

$$A_0 = A_1 = A_2 = A_3 \quad .$$

In consequence, jointly with the fact that data is given at different height levels (and movements through them), this tells us that our SCB dynamics in the crypt takes place over four levels of the same size (i.e., the same number of cells). Consistently, in order to derive theoretical predictions, we can project our dynamics into a 2D square-like surface with periodic boundary conditions, namely, *a cylinder.* In Extended Data Fig. 9d, we describe this mapping and in Extended Data Fig. 1b we provide the empirical number of Lgr5+ cells per level, which is fairly uniform and supports our approach.

1.3.2. *SCB over a 2D cylinder is formally equivalent to the 1D problem.*

We then need to apply the theory described above to the particular case of cylindrical geometry. In a cylindrical surface of radius R, we have that:

$$\sqrt{g} = R \quad .$$

In consequence, Eq. (1.15) reads:

$$S_n = \int_0^{2\pi} d\theta \int_{z=0}^{z_n} \sqrt{g} dz = 2\pi R z_n \quad ,$$

resulting into drift parameter $v(z)$, after application of Eq. (1.16):

$$v(z) = k_d z \quad ,$$

as in the 1D case. Therefore, *the SCB dynamics in a cylinder where the push up force is projected towards the z coordinate is formally equivalent to the 1D case.* Specifically, Eqs. (1.2, 1.10, 1.11, 1.12) and (1.13) have exactly the same form, simply considering the change $k_r \to k_r(z)$. The long-term dynamics is well fitted with predicted Gaussian distribution (see Fig. 2d and Extended Data Fig. 9e). Empirical ratios k_r/k_d have been fitted using long term data (56 days), as this is a very long time compared to the other timescales of the problem, allowing us to safely assume that this is within in the asymptotic regime governed by Eq. (1.13). Very good fits are obtained for both the large and SI using non-linear mean square fit provided by the standard `python` library `scipy.optimize`, leading to $k_r/k_d \approx 1.84$, with a mean square error of $\sim 10^{-2}$ for the SI and $k_r/k_d \approx 0.32$, with a mean square error of $< 10^{-5}$ for the LI.

Importantly, we also verified that numerical simulations and the theoretical derivation above agree very well with each, both for the steady-state survival probability and its temporal dynamics (Extended Data Fig. 9h,i), so that both can be used to fit the data of the 1D survival probability. For long-term monoclonal conversion however, the discrete simulations need to be used (see below for details) as monoclonal conversion is intrinsically a concept that requires a discrete finite system. We also use 2D simulations (see below) in the subsequent statistical analysis because it allows us more straightforwardly to derive non-parametric confidence intervals based on the amount of data collected in each experiment.

1.3.3. *Effective number of stem cells in 2D cylindrical geometry.*

Due to the 2D nature of the cylinder, the number of effective stem cells will differ from the 1D case by a geometrical pre-factor. In particular, we have to solve Eq. (1.19) considering a 2D cylindrical surface, with $\sqrt{g} = R$. According to the above reasonings, the scale of the (vertical) fluctuations will be given by $\sigma \sim \sqrt{\frac{k_r(z)}{k_d}}$. In consequence, If we consider 2σ's of representativeness, we will cover around 95.5% of the cases, leading the effective number of stem cells to be defined as:

$$N_{crypt} \approx 2\pi R \left(1 + 2\sqrt{\frac{k_r(z)}{k_d}} \right) \quad . \tag{1.20}$$

We identify $2\pi R$ as the perimeter of the crypt in units of cell length and, thus, the number of cells per level N_g. According to empirical observations (see Extended Data Fig. 1b), we assume that:

$$N_g \approx 5 \text{ cells} \quad ,$$

which leads to the estimation of effective number of stem cells, considering an amplitude of fluctuations of 2σ, to be:

$$N_{crypt} \approx 5 \left(1 + 2\sqrt{\frac{k_r(z)}{k_d}} \right) \quad .$$

1.4. **Nature of the stochastic rearrangements and retrograde movements.**

At the mathematical level, the parameter $k_r(z)$ describes fluctuations in the position of cells along the axis defined by the push-up force exerted by proliferation. However, this description remains agnostic as to the underlying nature of the rearrangements, both at the cellular and molecular levels. In consequence, $k_r(z)$ can be the outcome of several processes, whose only common ingredient is that they result in stochastic fluctuations in the position of cells along the crypt-villus axis. We discuss some possibilities below.

1.4.1. *Relation between retrograde and random movements.*

We consider that the cells populating the 2D surface of the crypt perform stochastic rearrangements in four generic directions: up, down, left and right, at rate k_r. "Left" and "right" movements are irrelevant from the point of view of the model, because they do not change the favourability of the cell position. If the direction taken by the cell is "down", we call this movement "retrograde movement", as this is the key process that can lead to non-trivial competition (in its absence, only the first row of cells compete on the long-term). However, because of the requirement of constant cellular density, any "retrograde" movement downwards from a given cell has to be compensated by an upwards movement from another cell. Therefore, even in scenarios where stem cells in SI actively migrate actively towards the bottom of the crypt (consistent with the experimental findings of Fig. 3), the constraint imposed by homeostasis (constant cellular density) implies that the net dynamics of the retrograde movements will result into stochastic rearrangements.

1.4.2. *Epithelial "tectonic" movements.*

In the simulations, we assume that the stochastic re-arrangements are only between nearest neighbours, which switch position. However we could also imagine more collective modes of re-arrangements, i.e. "tectonic" movements. By "tectonic" movement, we denote global rearrangements of the epithelium relative to the bottom of the crypt. Numerical simulations show that such (stochastic) tectonic movements are also well-described in our coarse-grained model, simply by renormalizing the effective rate of rearrangements k_r over the long term [5]. However, one would expect tectonic movements to change the short-term dynamics: If stochastic rearrangements come from tectonic movements, one does not expect clonal dispersion for instance, despite multiple cell rows being able to contribute long-term.

2. Numerical simulations of the 2D SCB dynamics and statistics

Although the analytical arguments above provide accurate predictions for key observables arising from the stochastic conveyor belt dynamics (along the crypt-villus axis), such as the evolution of the survival probability as a function of starting position, they do not take into account the (finite) circumferential size of the crypt. This is necessary in particular to understand metrics such as monoclonal conversion time. Past models have concentrated on this aspect by modelling the neutral dynamics of lateral competition along a 1D ring of equipotent stem cells [1, 2, 3], which leads to a diffusive process of monoclonal fixation. However, combining the two dynamics is highly non-trivial in the general case, leading us to rely on 2D numerical simulations for parameter-fitting and predictions.

2.1. Details on the implementation of 2D simulations.

Simulations are done on a square lattice with periodic boundary conditions in order to reproduce a cylindrical geometry. Such a cylinder consists of L rows and N_g cells per row, so that we define cell position by the coordinate (i, j), where $i \in [0, L]$ and $j \in [1, N_g]$ (periodic boundary condition used along the j direction). Based on the data, we take $N_g = 5$ (see Extended Data Fig. 1b), whereas the number L of rows chosen is largely irrelevant (as shown in the sections above, as long as $L \gg \sqrt{k_r/k_d}$) so we take L=20 in the simulations. We define the crypt region as the first 4 rows based on the pattern of Lgr5+ expression observed *in vivo*, so that the simulations are initialized at $t = 0$ by labelling a cell at a random position in the first 4 rows (20 positions possible).

Cells divide as a Poisson process at rate k_d, in a spatially isotropic manner: 50% of divisions occur along the crypt-villus axis, so that a cell at position (i, j) produces an identically labelled cell at position $(i+1, j)$, and displaces all cells of column j above it by +1. The rest of the divisions occur laterally, so that a cell at position (i, j) produces an identically labelled cell at position $(i, j-1)$ or $(i, j+1)$ and the cell that was at this position is expelled along side its column to a position $(i+1, j-1)$ or $(i+1, j+1)$ (see Fig. 2e for a sketch).

Moreover, we also assume that cells can reposition to different rows, as a Poisson process at rate k_r. As discussed above, several microscopic implementations of this process can be envisioned, but give rise to similar behavior, so that we implement the simplest version: repositioning exchanges the positions of a cell at position (i, j) with one of its neighbours of the same column, either $(i+1, j)$ or $(i-1, j)$. We note that we could also implement a process of dispersion/repositioning along the lateral direction, but i) such a process is neutral, in the sense that it does not contribute to repositioning towards the center of the niche, and ii) little clonal dispersion is observed in the lateral direction *in vivo*, arguing against a strong prevalence of such events.

2.2. Statistical approach for fitting and confidence intervals.

As we have shown analytically, the probability of lineage survival $p(c_n)$ as a function of starting position n of the mother cell rapidly converges towards a Gaussian form, despite its amplitude decreasing over time as monoclonal conversion occurs. For fitting the long term (8-week intravital) tracing data, it is thus convenient

to normalize the amplitude of $p(c_n)$ by its sum over all c_n (which becomes the probability of a a cell at a given starting site winning the competition compared to other site, rather that the fraction of surviving clones at a given site, among those that started at the same site).

We thus ran 2D numerical simulations for $T = 56$ days, simulating 10000 clonal labelling events, and using a division rate of $k_d = 5$ per week (although as mentioned above, the exact value of the division rate irrelevant for the fit to long-term normalized survival distributions). We performed a parameter sweep $k_r/k_d \in [0, 10]$, and used a least-squares method on the normalized survival probability distribution of the 8-week intravital tracing (see Fig. 2d), to identify the best-fit value of k_r/k_d in both SI and LI. For completeness, we have also performed a sensitivity analysis where we report the behavior of the model for several time points when varying independently the values of k_r and k_d (Extended Data Fig. 9h).

To build confidence intervals in a non-parametric way, we used a bootstrapping with replacement approach. The SI and LI data consisted respectively of 268 and 294 clones across starting positions $i = 0, 1, 2, 3$, so we re-sampled the dataset 1000 times for both LI and SI, and performed a least-squares fit to the theoretical expectation for each re-sampled dataset. This allowed us to build 95% confidence intervals together with best-fit values (which are reported in the main text).

We found that in both SI and LI, the predictions using the best fit values of k_r/k_d captured well the gradual decay of survival probability with position observed in the data (Fig. 2d). The shaded intervals shown were obtained by running the model with the best-fit value only, but simulating only the observed number of clones (resp. 323 and 351), and calculating 1 standard-deviation confidence intervals, to give a feeling for the influence of finite sampling of the model prediction.

With the best-fit values extracted via the long-term survival probability, we then tested whether the model could predict the short-term evolution of the survival probability as a function of starting position. Unlike its long-term counterpart, this transient does depend on the absolute value of the division rate k_d, which sets the time-scale of the problem. However, this value is heavily constrained by past cell kinetics measurements from the literature, which have measured a typical division time of 1.2 days at homeostasis [1, 2]. This is also consistent with our own measurements of $f_b = 20 - 25\%$ of cells labelled by a short EdU pulse (Fig. 1k,l). Indeed, given prior estimates of an S-phase duration of $T_s = 7.5h$ being highly conserved [11] would translate into a division time T_s/f_b between 1.2 and 1.5 days. We note that although we find small statistical differences between the proliferation rates of SI and LI (Fig. 1l), these were very small compared to the very large value of the difference between k_r in both regions (see below for more details). Together with our data on the role of Wnt-dependent active migration in setting the value of k_r, this argues that the main difference between SI and LI is due to active retrograde movements.

2.2.1. *LI.*

We first started with the short-term LI data, and found that using a value of division time of 1.2 days, together with $k_r/k_d = 0.25$ (95% CI: 0.05-0.55) yielded good predictive power. To quantify this, we calculated an average residual $s = \sqrt{\sum_i^n r_i^2/n}$, where r_i is the difference between theoretical (best-fit value) and experimental persistence, for day 2, 3, 4 and 56, and for position 0,1,2 and 3 (so that we sum over n=16

data points), and found $s = 0.07$, indicative of a good fit. We then performed a parameter sweep over k_d, to check that the fit was not improved by choosing a different value of division time, and found an optimum fitting value of a division every 1.4 days, within the range of value inferred from EdU pulse experiments (see above), which only marginally improved the fit ($s = 0.06$). This demonstrates the predictive power of the theory. Computing as above the confidence interval on the prediction with the best-fit value, but finite statistics (number of crypts considered in the respective experiment, shaded area in Fig. 3g, Extended Data Fig. 9i,j,k) showed that all data points fell within the prediction.

Furthermore, setting the division rate to once every 1.4 days, and performing a parameter sweep to fit k_r/k_d from the short-term data only (day 2, 3 and 4) led to a best-fit value of $k_r/k_d = 0.4$ (95% CI: 0-1), fully in line with the value fitted from the long-term persistence. From the same data and simulations, we could also extract the transfer probability between center and border rows in crypts (Extended Data Fig. 9l,m). To do this, we calculate (both in simulations and data, at day 2, 3 and 4 of the tracings) the probability for clones that started at the border (resp. at the center) at day 1 to be either i) lost from the stem cell region, ii) only in the crypt border, or iii) have some cells in the crypt center. Again, we found consistently good agreement between data and the model (using the same values as above).

2.2.2. *SI.*

Using the same approach as in the LI, for the SI we found best value $k_r/k_d = 2$ extracted from the long-term SI data. As expected from the higher clonal dispersion, we observed in the data a much larger probability for border cells to go back to the center of the crypt (Fig. 3e), and consequently a weaker gradient of clonal loss as a function of starting position (see Extended Data Fig. 9h,j,k). Screening again for optimal values of division rate allowed for a very good fit ($s = 0.07$) of the survival probability as a function of time and starting position (see Extended Data Fig. 9j). However, this optimal fit was achieved for a slower division rate than inferred from EdU (division time of 2.8 days). This initially puzzling feature came from the fact that persistence is globally higher (on average over all positions) than in LI, which cannot be explained by changes in k_r. One possibility could be that the method of observation (and presence of an imaging window) causes the dynamics to be slower than expected on short-time scales in SI. Alternatively if we constrain the division rate to be the rate as LI, a way to explain the discrepancy is to assume that the labelling method labels preferentially cells about to undergo mitosis: if we initialise the simulation by 2 cells instead of 1 in the SI, then we can achieve the best-fit of the short-term data ($s = 0.07$) for the same division rate as SI (one division every 1.4 days).

We then turned to the transfer probability between center and border in small intestinal crypts. As expected, the ratio of starting border cells making it to the center regions to those being lost was much higher in SI than LI (0.18 in LI vs 0.57 in SI at day 2, 0.02 in LI vs 0.19 in SI at day 3, 0.2 in LI vs 0.18 in SI at day 4), which is in very good quantitative agreement with our model predictions (see Fig. 3g,h, Extended Data Fig. 9l,m)

2.2.3. *SI with LGK974 inhibitor.*

Next, we sought to challenge the model further by confronting it to perturbation experiments. We thus re-analyzed published data [12] on stem cell dynamics in the small intestine upon LGK974 treatment (which has been shown to accelerate monoclonal conversion) within the framework of our model, as well as performed new genetic experiments using APC hypomorph mice.

In LGK974 treatment condition, as we do not have access to the long-term persistence as a function of starting position, we set the division rate to once every 1.4 days, and proceeded as above to fit k_r/k_d based on the short-term persistence as a function of starting position (at day 2, 3 and 4). Interestingly, we found that $k_r/k_d = 0.4$ (95% CI: 0-1, a similar value to the one extracted in wild-type LI), provided again a good fit for the dataset (see Fig 3g). We note that in this experimental condition, we could explain the dynamics of the data with only one initially labelled cell, although further work should be performed to understand how the condition changes exactly the initial labelling of the system. Overall, this analysis argues that LGK974 treatment nearly abolishes the retrograde movements observed in normal small intestinal crypts, which we observe experimentally (see Fig. 3e), making the dynamics closely resemble the one of large intestinal crypts.

Similarly, turning to the transfer probability between centre and border, we found that border cells nearly never returned to central position over the time course of the live-imaging (occurring at a single day for a single clone, out of 19 clones imaged at days 2, 3 and 4), and that the full transfer probabilities in time were well-captured by the model (see Fig. 3h).

2.3. Clonal fragmentation from fixed data.

Another way of independently testing the model was to test whether the k_r/k_d parameter that we extracted from the long-term clonal survival experiments could recapitulate not only the short-term survival dynamics (section above), but also the spatial profiles and shapes of clones at a fixed time point. Intuitively, large values of k_r/k_d should give rise to extensive clonal fragmentation, which should manifest as non-cohesive clones (Extended Data Fig. 10a).

We reasoned that such differences should be most manifest for intermediary time points: at very late time points, clones occupy a significant fraction of the crypts, and extend along the entire crypt-villus axis, so that fragmentation will be "geometrically" reduced, while for very early time points, there are too few cells to observe fragmentation. We thus settled experimentally on day 7 post-clonal labelling (Extended Data. Fig. 10b), and we systematically assessed the number of clones which were cohesive or non-cohesive in LI vs SI (we defined a non-cohesive clone as one were a gap of at least one non-labelled cell was observed between two clonal fragments along the crypt-villus axis).

Importantly, we found clear differences between SI and LI in the experimental data (Extended Data Fig. 10b,c): whereas $66\% \pm 20\%$ of clones showed fragmentation in SI, this was only the case for $21\% \pm 16\%$ of clones in LI (we indicate mean $\pm$ SD). This validates qualitatively the hypothesis of higher values of random relocation movements k_r in small intestine, in an independent manner.

Furthermore, to test whether this could also be recapitulated quantitatively, we ran the same model as above for SI and LI for 7 days, using the exact same values of $k_r/k_d = 2$ and $k_r/k_d = 0.25$ respectively. Interestingly, defining clonal fragmentation in the same way (one non-labelled cell between two labelled cells

along the crypt-villus axis), we found that the simulations predicted 27% of fragmentation in LI and 59% in SI (Extended Data Fig. 10c), in good agreement with the data.

2.4. **Timescale of monoclonal conversion.**

Finally, we sought to test whether the model could also capture the long-term conversion towards crypt monoclonality that is observed in static lineage tracing experiments. Such experiment provides an independent test of our framework, all other parameters having been measured or fitted as previous described. Furthermore, we also provide a systematic theoretical analysis for the model expectation across all values of k_r

For $k_r = 0$, the neutral 1D dynamics of [1, 2] holds, so that crypts drift to monoclonality on the characteristic time scale of $T_m \approx \frac{N_g^2}{k_d}$. We then sought to compute T_m in our stochastic conveyor belt model, as a function of k_r/k_d and N_g. We ran the same dynamics as described above, and defined crypts as monoclonal when all cells in the first 4 rows were labelled. We then computed the evolution of the average fraction of monoclonal crypts over time. Interestingly, using values of $k_r/k_d = 0.25$ (LI) and $k_r/k_d = 2$ (SI), together with the same division rate as before (division every 1.4 days) yielded an excellent quantitative agreement with experimental data (see Fig. 4c). For LI, the 1D ring model predicts $T_m \approx \frac{N_g^2}{k_d} \approx 5w$, close to the observed dynamics in 2D (which is expected for such low k_r), where the dynamics is significantly slower for SI.

To make this more systematic, we simulated the monoclonal conversion for a large range of parameters $k_r/k_d \in [0, 20]$. Interestingly, we found that the monoclonal conversion curves for different values of k_r (see Extended Data Fig. 9f,g) could be rescaled on each other, and that the rescaled monoclonal time was well-fitted by a square-root dependency $T_m(k_r)/T_m(k_r = 0) \approx \sqrt{k_r}$ (see Extended Data Fig. 9f,g), arguing that monoclonal conversion occurs on timescales quadratic in N_g, and quasi-linear in N_s.

Interestingly, small intestinal crypts in the presence of the LGK inhibitor drift to monoclonality even faster than expected from $N_g = 5$ and $k_r = 0$, even though the cell division rate was reportedly unaffected [12]. However, for very small values of k_r, our definition of discrete cell rows (which arises from projecting SCB dynamics from a half-sphere to a cylinder) becomes too strong. Indeed, the LGK monoclonal conversion time scale can be well-fitted by a model where $k_r \approx 0$ and $N_g = 3$ (see Fig. 4d), arguing that retrograde movements become so small that only certain cells at the very "bottom" of row 0 can now win the competition over the long-term.

2.5. **Dynamics of filling after ablation.**

Simulations of the SCB dynamics in regenerative scenarios after ablation were performed assuming, as initial condition, that the crypt is empty at levels (0,+1,+2,+3), but that an additional level, +4, is populated by LGR5+ cells. These cells, in turn, divide at rate k_d and perform retrograde movements at rate $\hat{k}_r$. If a duplication event at position j happens such that either the position $j-1$ or the position $j+1$ is empty, the newborn cell occupies position $j-1$ or $j+1$ respectively. If both positions $j-1$ and $j+1$ are empty, the newborn cell choses one of the two positions, either $j-1$ or $j+1$, at random, with equal probability.

If a cell at position j undergoes a retrograde movement towards position $j-1$, just moves to that position, if such a position is empty, or exchanges its position with the cell located at $j-1$. For the sake of clarity, we write $\hat{k}_r$ instead of k_r as we did in confluent tissue at homeostasis. The reason lies in the potential existence of gaps in the epithelium in the regeneration scenario, leading to situations in which a retrograde movement does not imply an exchange of positions, i.e., it does not trigger a random fluctuation, but a net downwards movement. In addition, one expects, specially at the beginning of the regeneration process, that the mechanical properties of the tissue may be different from the ones found for a confluent tissue in homeostasis. It is worth to note that this dynamics converges to the standard SCB dynamics we described so far as soon as the tissue becomes confluent.

Starting from an empty crypt as described above, we run the SCB dynamics and we track the position of the lowest cell in time. As expected, for comparable k_d values, the larger $\hat{k}_r$, the faster is the occupation of the lower levels –see Fig. 4h. This qualitative prediction of the model allows us to test the behaviour of the regenerative dynamics. From real data we extract the position of the bottom-most LGR5+ cell for days 2,4,7 and 15 after ablation –see Fig. 4i. Assuming $k_d \approx 0.5$, –i.e., ~ 1 division every two days–, we fitted the positions of the bottom-most cells in these sequence of days with a single value of $\hat{k}_r$. Results show a good qualitative agreement with data along the time sequence –see Fig. 4h. Best fits give us a $\hat{k}_r^{SI} \approx 5\hat{k}_r^{LI}$, fully compatible with the other results corresponding to confluent tissues at homeostasis shown in this study.

3. Comparison to previous models

Previous studies performing lineage-tracing in intestinal crypts had identified a faster drift to monoclonality in large compared to SI (around 3-6w vs 8w for half the crypts to become monoclonal) [1, 2]. This was also found using a continuous labelling approach, where the time scale for monoclonality can be inferred by comparing the fraction of partially labelled to fully labelled crypts [3], and where colon was found to drift to monoclonality 2-3 times faster than SI. In this latter study, this difference was ascribed to differences in the symmetric division rate λ_{sym} of stem cells in SI (1-2 symmetric divisions a week) versus LI (3 symmetric divisions a week), with functional stem cell numbers being similar in both regions ($N_s = 5-7$ per crypt). Given the fact that the stem cell division rate λ is close to once a day in both regions, this would mean that most divisions in SI are asymmetric (at a rate λ_{asym}). This also goes against our findings from live-imaging of a larger number of effective stem cells in SI compared to LI.

However, Ref. [3] makes three important assumptions: i) N_s compete neutrally along a 1D ring (which leads to monoclonal conversion on the time scale of N_s^2/λ_{sym}, ii) continuous labelling (arising in the case of spontaneously occurring frameshift mutations) occurs at the same rate α in both small and LI, and iii) seemingly, only symmetric divisions can give rise to a mutation that leads to labelling, so that the rate of monoclonal crypt labelling is:

$$\Delta C_{fix} = \alpha\lambda_{sym} \quad . \tag{3.1}$$

Assumption ii) and iii) are critically important, because the differences of symmetric division rate λ_{sym} between SI and LI reported in Ref. [3] then arise directly from observed differences in ΔC_{fix}. However, little evidence of intrinsically asymmetric division exists in intestinal crypts, and no "immortal strand" has

been observed upon division: thus labelling events should also occur upon asymmetric division event, and the 3-fold differences observed in symmetric division rate ΔC_{fix} can only arise from difference in absolute division rate (which does not seem to be the case between SI and LI) or labelling rate α. Given that ΔC_{fix} is observed to be 2.8 times larger in LI compared to proximal SI, we can then infer that α should be 2.8 times larger also in LI. Importantly, α also enters into the fraction of partially labelled crypts [3]:

$$C_{part} = \alpha N_{crypt}(N_{crypt} - 1)/2 \quad , \tag{3.2}$$

which is used in Ref. [3] to deduce the number of stem cells per crypt N_{crypt} in both regions. Correcting for α can then be used to predict stem cell numbers. However, Eq. 3.2 critically depends on assumption i), i.e.dynamics of stem cells along a 1D ring, and would take a different form in different geometries [13].

In the limiting case $k_r/k_d \approx 0$ (which is relevant for LI from our live-imaging data), only a single row of stem cells contributes, so our data supports $N_{crypt} \approx 7$. This is in close agreement with the findings of Ref. [3] in colon, which is expected because Eq. 3.2 holds in this effectively one-dimensional situation (with $C_{part} = 2.29 \times 10^{-5}$), and if half of divisions are lateral, a global division rate of once every 1-1.5 day translates to a symmetric division every 2-3 days as assumed in [3]. In SI, fitting as discussed above from ΔC_{fix} that $\alpha_{LI} = 2.8\alpha_{SI}$, we can still first apply the 1D ring model (Eq. 3.2, with $C_{part} = 1.32 \times 10^{-5}$ in proximal SI), which then predicts a slightly larger stem cell number in SI of $N_{crypt} \approx 9$. However, in this case, the 1D ring approximation starts losing its validity, since we find in our live-imaging data that $N_s \approx 2.5$ (which leads to $N_{crypt} \approx 12$).

In the general case, one should note the definition of the stem cell number (and how it influences the timescale for monoclonality T_m) differs between the SCB dynamics model and the 1D ring model. Getting such a simple analytical approximation for C_{part} is more difficult, in particular because there are two length scales defining stem cell number $N_{crypt} = N_g N_s$: the number N_g of stem cells per row (defined by tissue geometry) and the number N_s of rows participating to the competition of the SCB dynamics. As discussed above however, the time to monoclonality T_m scales in a different manner with N_g and N_s, with a quadratic scaling in N_g as in the 1D ring model, but a quasi-linear scaling in N_s. This is consistent with the fact that SI drifts to monoclonality around 1.6 times slower than LI (given that we find N_s in SI around twice larger than in LI). This is also consistent with the ratio of partial crypts in SI vs LI found in Ref. [3], when correcting for the different mutation rate: $\frac{C^{SI}_{part}\alpha_{LI}}{C^{LI}_{part}\alpha_{SI}} \approx 1.6$.

Supplementary references

[1] Lopez-Garcia, C., Klein, A.M., Simons, B.D. and Winton, D.J., 2010. Intestinal stem cell replacement follows a pattern of neutral drift. Science, 330(6005), pp.822-825.

[2] Snippert, H.J., Van Der Flier, L.G., Sato, T., Van Es, J.H., Van Den Born, M., Kroon-Veenboer, C., Barker, N., Klein, A.M., Van Rheenen, J., Simons, B.D. and Clevers, H., 2010. Intestinal crypt homeostasis results from neutral competition between symmetrically dividing Lgr5 stem cells. Cell, 143(1), pp.134-144.

[3] Kozar, S., Morrissey, E., Nicholson, A.M., van der Heijden, M., Zecchini, H.I., Kemp, R., Tavarè, S., Vermeulen, L. and Winton, D.J., 2013. Continuous clonal labeling reveals small numbers of functional stem cells in intestinal crypts and adenomas. Cell stem cell, 13(5), pp.626-633.

[4] Ritsma, L., Ellenbroek, S.I., Zomer, A., Snippert, H.J., de Sauvage, F.J., Simons, B.D., Clevers, H. and van Rheenen, J., 2014. Intestinal crypt homeostasis revealed at single-stem-cell level by in vivo live imaging. Nature, 507(7492), pp.362-365.

[5] Corominas-Murtra, B., Scheele, C.L.G.J., Kishi, K., Ellenbroek S.I.J., Simons, B. D., van Rheenen, J., and Hannezo, E., 2020. Stem cell lineage survival as a noisy competition for niche access. 2020 Proceedings of the National Academy of Sciences USA 117 (29), pp.16969-16975

[6] Karatzas, I., and Shreve, S. E., 1988 *Brownian Motion and Stochastic Calculus* Springer-Verlag:Berlin.

[7] Gardiner, C. W., 1983 *Handbook of Stochastic Methods for Physics, Chemistry and the Natural Sciences* Springer-Verlag:Berlin.

[8] Van Kampen, N. G., 2007 *Stochastic Processes in Physics and Chemistry* third edition, North Holland:Amsterdam.

[9] W. Klingenberg (1978) *A course on differential geometry* Translated by D. Hoffman. Springer-Verlag:Berlin.

[10] W. Kühnel (2006) *Differential Geometry: Curves–Surfaces–Manifolds* 2nd Edition. (Student Mathematical Library series, 77) American Mathematical Society:New york.

[11] Itzkovitz, S., Blat, I. C., Jacks, T., Clevers, H., and van Oudenaarden, A. 2012 Optimality in the development of intestinal crypts. Cell, 148(3), 608-619.

[12] Huels, D.J., Bruens, L., Hodder, M.C., Cammareri, P., Campbell, A.D., Ridgway, R.A., Gay, D.M., Solar-Abboud, M., Faller, W.J., Nixon, C. and Zeiger, L.B., 2018. Wnt ligands influence tumour initiation by controlling the number of intestinal stem cells. Nature communications, 9(1), pp.1-10.

[13] Klein, A.M. and Simons, B.D., 2011. Universal patterns of stem cell fate in cycling adult tissues. Development, 138(15), pp.3103-3111.
